# CRISPR-mediated multigene integration enables Shikimate pathway refactoring for enhanced 2-phenylethanol biosynthesis in *Kluyveromyces marxianus*

**DOI:** 10.1186/s13068-020-01852-3

**Published:** 2021-01-06

**Authors:** Mengwan Li, Xuye Lang, Marcos Moran Cabrera, Sawyer De Keyser, Xiyan Sun, Nancy Da Silva, Ian Wheeldon

**Affiliations:** 1 Department of Chemical and Environmental Engineering, University of California Riverside, Riverside, CA 92521 USA; 2 Department of Chemical and Biomolecular Engineering, University of California Irvine, Irvine, CA 92697 USA; 3 Center for Industrial Biotechnology, University of California Riverside, Riverside, CA 92527 USA

**Keywords:** Metabolic engineering, Flavors and fragrances, Thermotolerance, Expression regulation

## Abstract

**Background:**

2-phenylethanol (2-PE) is a rose-scented flavor and fragrance compound that is used in food, beverages, and personal care products. Compatibility with gasoline also makes it a potential biofuel or fuel additive. A biochemical process converting glucose or other fermentable sugars to 2-PE can potentially provide a more sustainable and economical production route than current methods that use chemical synthesis and/or isolation from plant material.

**Results:**

We work toward this goal by engineering the Shikimate and Ehrlich pathways in the stress-tolerant yeast *Kluyveromyces marxianus*. First, we develop a multigene integration tool that uses CRISPR-Cas9 induced breaks on the genome as a selection for the one-step integration of an insert that encodes one, two, or three gene expression cassettes. Integration of a 5-kbp insert containing three overexpression cassettes successfully occurs with an efficiency of 51 ± 9% at the *ABZ1* locus and was used to create a library of *K. marxianus* CBS 6556 strains with refactored Shikimate pathway genes. The 3^3^-factorial library includes all combinations of *KmARO4*, *KmARO7*, and *KmPHA2*, each driven by three different promoters that span a wide expression range. Analysis of the refactored pathway library reveals that high expression of the tyrosine-deregulated *KmARO4*^*K221L*^ and native *KmPHA2*, with the medium expression of feedback insensitive *KmARO7*^*G141S*^, results in the highest increase in 2-PE biosynthesis, producing 684 ± 73 mg/L. Ehrlich pathway engineering by overexpression of *KmARO10* and disruption of *KmEAT1* further increases 2-PE production to 766 ± 6 mg/L. The best strain achieves 1943 ± 63 mg/L 2-PE after 120 h fed-batch operation in shake flask cultures.

**Conclusions:**

The CRISPR-mediated multigene integration system expands the genome-editing toolset for *K. marxianus,* a promising multi-stress tolerant host for the biosynthesis of 2-PE and other aromatic compounds derived from the Shikimate pathway.

## Background

Like many esters and alcohols produced during yeast fermentation, the aromatic alcohol 2-phenylethanol (2-PE) is used in a wide variety of applications. Its rose-like aroma is used to add flavor and fragrance to food, perfumes, and cosmetics [1]. The high energy density and compatibility with gasoline blends also makes 2-PE a promising next-generation biofuel [2]. The worldwide flavor and fragrance market was valued at upward of $US20 billion in 2018 and is expected to expand over the next decade (https://www.grandviewresearch.com/industry-analysis/flavors-fragrances-market) [3]. High purity, food-grade 2-PE is still produced mainly by isolation from rose petals, which presents a technically challenging separation problem and the supply is subject to annual fluctuations in crop yields [4]. The end result is a high market price of ~ $US1000 per kg. Synthesis by chemical catalysis is possible, but typically produces 2-PE that sells for less than $US4 per kg because it is not suitable for human consumption or use [5]. The chemical catalysis route also creates environmental and health challenges due to the reliance on petrochemical reagents [6]. As a metabolic intermediate formed via the Shikimate and Ehrlich pathways, 2-PE produced by microbial systems is a potentially sustainable alternative to fossil fuel-based production and isolation from native plants.

**Fig. 1:**
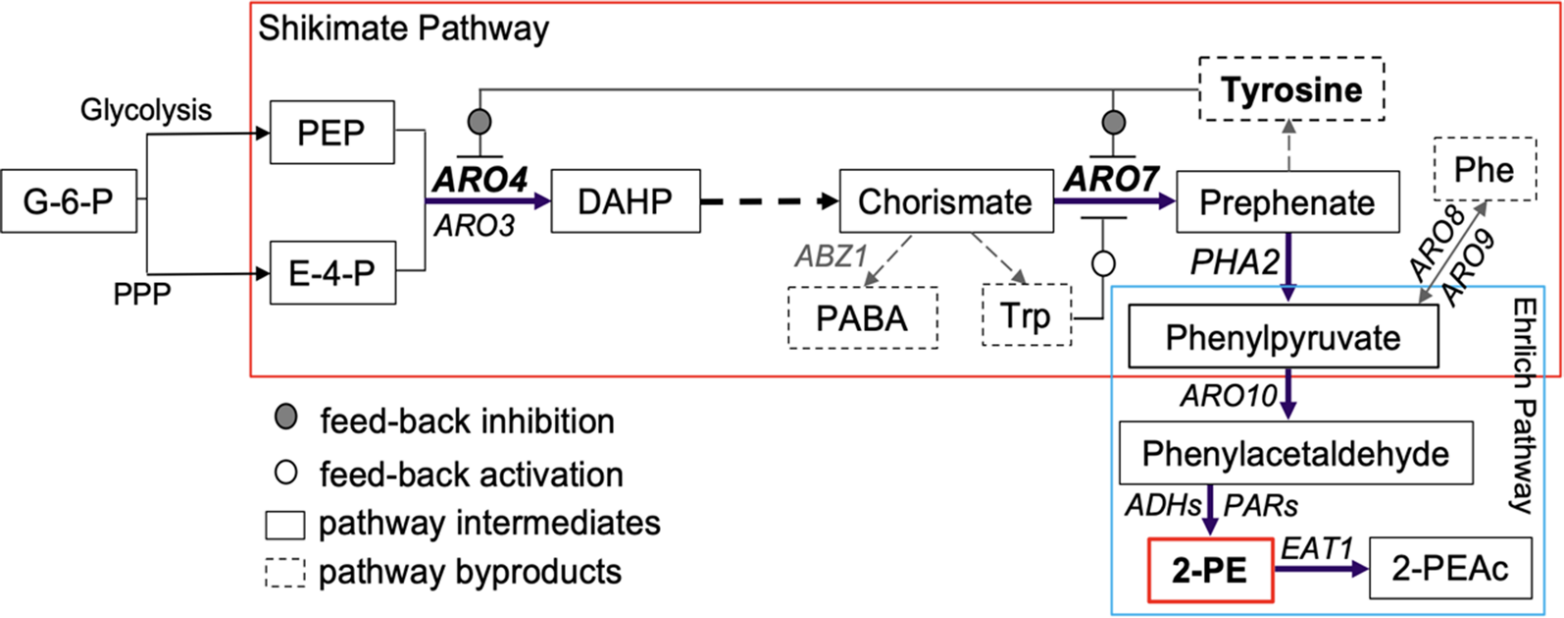
Simplified diagram of the Shikimate and Ehrlich pathways in *K. marxianus* for 2-phenylethanol biosynthesis. Arrows with dashed lines represent more than one reaction. *G-6-P*: glucose 6-phosphate; *PEP*: phosphoenolpyruvate; *E-4-P*: erythrose-4-phosphate; *DAHP*: 3-deoxy-d-arabinoheptulosonate-7-phosphate; *PABA*: para-aminobenzoate; *2-PE*: 2-phenylethanol; *2-PEAc*: 2-phenylethyl acetate. *ARO3/ARO4*: DAHP synthase; *ABZ1*: PABA synthase; *ARO7*: chorismate mutase; *PHA2*: prephenate dehydratase; *ARO8/ARO9*: aromatic aminotransferase; *ARO10*: phenylpyruvate decarboxylase; *ADH*: alcohol dehydrogenase; *PAR* phenylacetaldehyde reductase; *EAT1*: ethanol acetyltransferase

The Shikimate pathway is broadly conserved across various plants and microbes, producing aromatic amino acids from erythrose-4-phosphate (E4P) and phosphoenolpyruvate (PEP; Fig. 1). Chorismate is the first branch point, with one branch leading to tryptophan and the other to prephenate. One pathway output from prephenate is l-tyrosine, synthesized by prephenate dehydrogenase and aromatic aminotransferase. l-phenylalanine biosynthesis begins with prephenate as well, but prephenate is first converted to phenylpyruvate (PP) prior to transamination. Decarboxylation of phenylpyruvate, the beginning of the Ehrlich pathway, produces phenylacetaldehyde, with subsequent catalysis by aromatic alcohol dehydrogenase resulting in 2-PE. Acetylation synthesizes the corresponding ester, 2-phenylethyl acetate (2-PEAc), by alcohol acetyltransferase (ATTase or Atf) [7–9]. These biosynthetic pathways have been exploited to overproduce both 2-PE and 2-PEAc. Relieving tyrosine feedback inhibition at key nodes in the Shikimate pathway has produced substantial gains in 2-PE and 2-PEAc titers [10]. Overexpression of homologous and heterologous genes of the Ehrlich pathway, including phenylpyruvate decarboxylase and alcohol dehydrogenase (or phenylacetaldehyde reductase, PAR) is known to increase titer [11–13]. Finally, in situ product removal also has been shown to increase 2-PE titers beyond 2 g/L by alleviating product toxicity [14].

Many of the Shikimate and Ehrlich pathway metabolic engineering studies used the common yeast *Saccharomyces cerevisiae* as a production host [15–17], but the pathways have also been engineered in non-conventional yeasts such as *Yarrowia lipolytica*, *Scheffersomyces stipitis*, *Pichia pastoris*, and *Kluyveromyces marxianus* [18–21]. In our own work, we have been studying and engineering *K. marxianus* because wild type strains synthesize the short-chain, volatile ester ethyl acetate in grams per liter quantities, and medium-chain esters such as isoamyl acetate and 2-PEAc are produced in tens of milligram per liter quantities without metabolic engineering [8]. *K. marxianus* is classified as a generally regarded as safe (GRAS) organism, is thermotolerant to ~ 50 °C, and can metabolize various C5, C6, and C12 sugars [22–25]. Notably, *K. marxianus* is also one of the fastest-growing eukaryotes, with a maximum growth rate nearly twice that of *S. cerevisiae* at 30 °C under glucose limiting conditions [26, 27]. Finally, *K. marxianus* can use some genetic parts, including promoters and terminators, from *S. cerevisiae*, both of which are part of the saccharomyces subgenera within hemiascomycetes [28, 29]. For all of these reasons, we elected to use *K. marxianus* as the host for 2-PE biosynthesis in this work.

As is common for many non-conventional yeasts, there has been substantially less effort put towards creating advanced genetic and metabolic engineering tools than there has been for *S. cerevisiae*. Targeted gene integration is hampered by a low capacity for homologous recombination (HR); however, the widespread adoption of type II CRISPR systems for genome editing has helped alleviate this problem [30]. Targeted gene disruption, integration, and regulation have been made possible by CRISPR-Cas9 and CRISPR activation/interference (CRISPRa/i) systems for various non-conventional yeasts [31–35]. CRISPR-based genome editing has also been demonstrated in *K. marxianus*, but efficient multigene integration for pathway refactoring [36, 37] has not yet been established [8, 27, 38, 39].

Here, we engineered new strains of *K. marxianus* CBS 6556 with increased flux along the Shikimate and Ehrlich pathways, and high titer biosynthesis of 2-PE. To accomplish this, we first developed a CRISPR-Cas9-mediated multigene integration system that enabled simultaneous integration of up to three expression cassettes into a single, targeted genomic locus. Multigene integration was used to create a 3^3^ combinatorial library with variable expression of three key Shikimate pathway genes, *ARO4*, *ARO7* and *PHA2*. The elimination of tyrosine feedback inhibition to *KmARO4* and *KmARO7*, the deletion of parasitic pathways including *KmEAT1* acetylation of 2-PE to 2-PEAc, and the overexpression of *KmARO10* belonging to the Ehrlich pathway resulted in enhanced 2-PE production. Given the broad temperature range of *K. marxianus*, we also explored the effects of increased temperature. The highest 2-PE titer was achieved at 30 °C, with high expression of *KmARO4*^*K221L*^, *KmPHA2*, and *KmARO10*, medium-level expression of *KmARO7*^*G141S*^, disruption of functional *KmEAT1*, and cultures operated in fed-batch mode.

## Results

### CRISPR-mediated one-step, multigene integration

Given the importance of the Shikimate pathway to 2-PE production, we sought to increase flux to phenylpyruvate (PP), the precursor to the Ehrlich pathway, via Shikimate pathway refactoring. To accomplish this in *K. marxianus*, we first needed to identify a series of variable strength promoters, and secondly, create a multigene integration tool. We recently designed and characterized a set of *K. marxianu*s promoters with a wide expression range under glucose metabolism, and here elected P_*KmTEF3*_, P_*KmPGK*_ and P_*KmTDH3*_ to access high, medium, and low levels of gene expression in the refactored library [40].

**Fig. 2:**
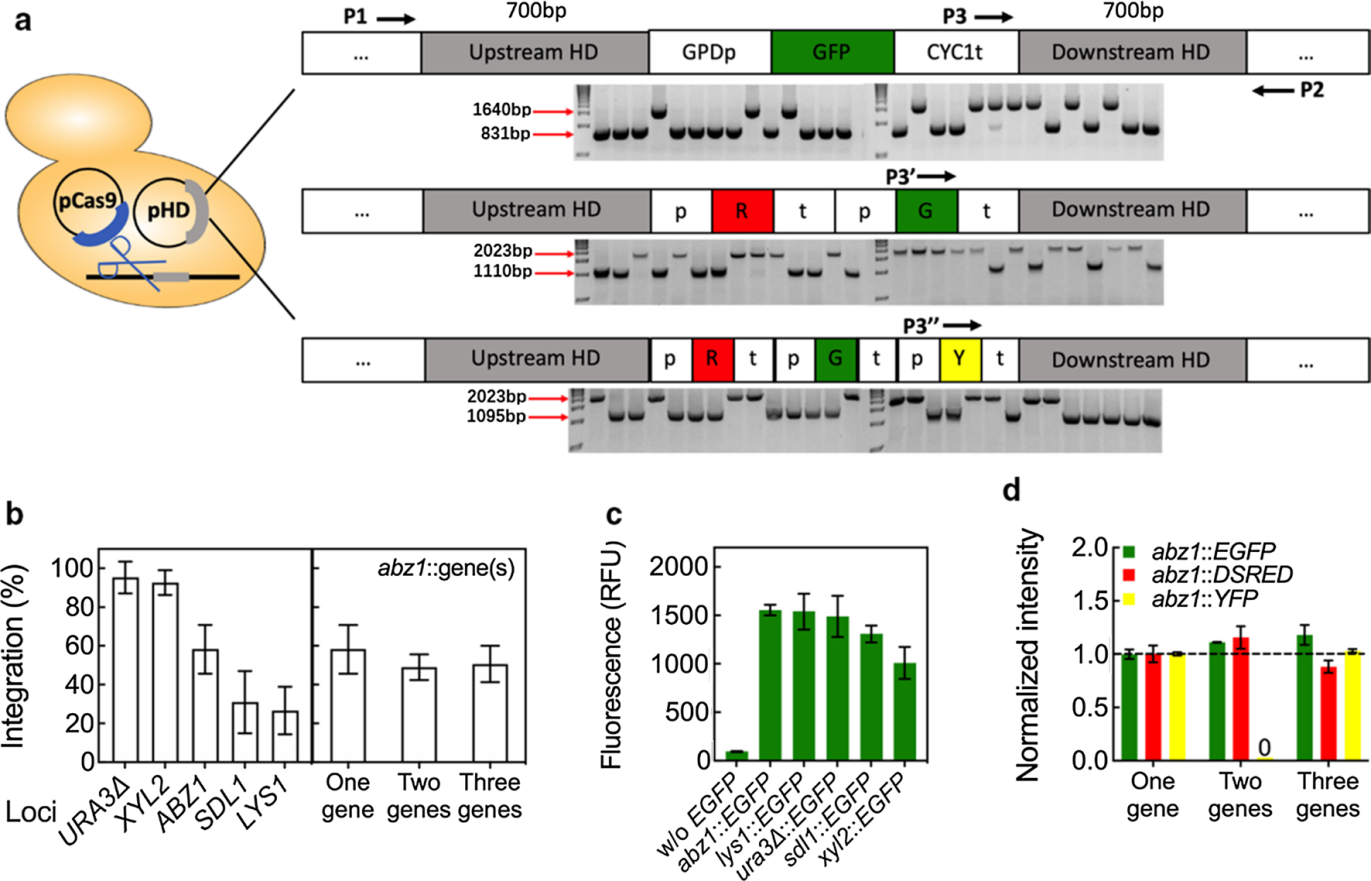
Markerless CRISPR-Cas9-mediated multigene integration in *K. marxianus* CBS 6556. **a** Schematic representation of the three-primer colony PCR test and example gels for single and multiple gene integrations into the *ABZ1* locus. The length of the up- and down-stream homology regions is 700 bp. **b** Integration efficiency of single (*EGFP*), dual (*EGFP*, *DSRED*), and triple (*EGFP*, *YFP*, and *DSRED*) genes. **c** Relative fluorescence intensity of *EGFP* expressed from different loci as measured by fluorescence microplate reader. **d** Normalized expression level of fluorescent proteins when produced individually or simultaneously with one or two additional fluorescent proteins. All experiments were performed in biological triplicate. Bars represent the mean, while error bars represent the standard deviation

Based on the design of a CRISPR-Cas9-mediated gene integration tool that we previously created for the oleaginous yeast *Y. lipolytica*, we designed a two-plasmid system for targeted gene integration in *K. marxianus* [41]. The CRISPR plasmid, pCRISPR, expressed a single guide RNA (sgRNA) along with Cas9. The homology donor plasmid, pHD, with 700 bp up- and down-stream homology to the targeted site was used for the integration of one, two, and three genes into a single locus. A schematic of the system and the homology donor constructs for single, dual, and triple gene integration are shown in Fig. 2a. Also shown is the three-primer verification test for chromosomal integration.

Prior to expanding the system for multiple genes, we identified five different genomic sites that were suitable for high-efficiency integration. *URA3*, *XYL2*, *ABZ1*, *SDL1* and *LYS1* were selected, and at least one sgRNA sequence that resulted in high-efficiency integration was identified. sgRNA sequences for each targeted gene are presented in Additional file 1: Table S1. The *URA3* locus was selected because wild type function was previously eliminated when creating the base strain *K. marxianus* CBS 6556 *ura3Δ his3Δ* [27]. *ABZ1* encodes aminodeoxychorismate synthase, which activates a parasitic reaction diverting chorismate to para-aminobenzoate (PABA; Fig. 1). *LYS1* was selected because it was previously identified as a hotspot for linear DNA integration [24], while *SDL1* is a non-essential gene in *K. marxianus*, and the gene knockout is insensitive to growth at elevated temperature [42]. Finally, *XYL2* was included in the set of potential integration sites because it facilitates rapid phenotypic screening for successful integrations and we have previously identified an sgRNA with high efficiency [27].

Heterologous gene integration at each of the selected loci was tested using fluorescent reporters *EGFP*, *YFP*, and *DSRED* (Fig. 2b). The highest integration efficiency of a single gene was 95 ± 8%, observed at the *URA3* locus. The second highest, 93 ± 6%, occurred at *XYL2*. For *ABZ1*, *SDL1* and *LYS1*, gene integration was achieved with efficiencies of 58 ± 13%, 31 ± 16%, and 27 ± 12%, respectively. Given these results, *ABZ1* was chosen as the primary site to develop the multigene integration system for Shikimate pathway refactoring because it is parasitic to flux along the pathway and because integrations were achieved with an efficiency of nearly 60%. Integration of an insert encoding expression cassettes for *EGFP* and *DSRED* occurred with 49 ± 7% of tested colonies, while an insert encoding all three reporter genes occurred at 51 ± 9% efficiency. Of note, shorter homology lengths down to 300 bp led to a 68 ± 11% decrease of integration efficiency (Additional file 1: Figure S1); therefore, 700 bp up- and downstream homology was used for all subsequent gene integrations.

Previous reports suggested that heterologous gene expression can differ depending on the integration site within the yeast genome [41, 43]. Figure 2c compares the *EGFP* fluorescence from integration at the five tested sites, including *ABZ1*, *LYS1*, *URA3*, *SDL1*, and *XYL2*. One-way ANOVA suggested that there was a significant effect due to integration locus, with the effect driven primarily from low expression at the *XYL2* locus (*p* = 0.0183, *n* = 3, Welch’s test). Expression from single, dual, and triple integrations was also tested (Fig. 2d). Based on fluorescence measurements, expression of a single gene was equivalent to the expression of the same gene when integrated along with other reporter genes at the same locus, thus providing a genome-editing tool that can be used to simultaneously refactor up to three genes of a desired pathway.

### Alleviation of feedback inhibition in the Shikimate pathway

**Fig. 3:**
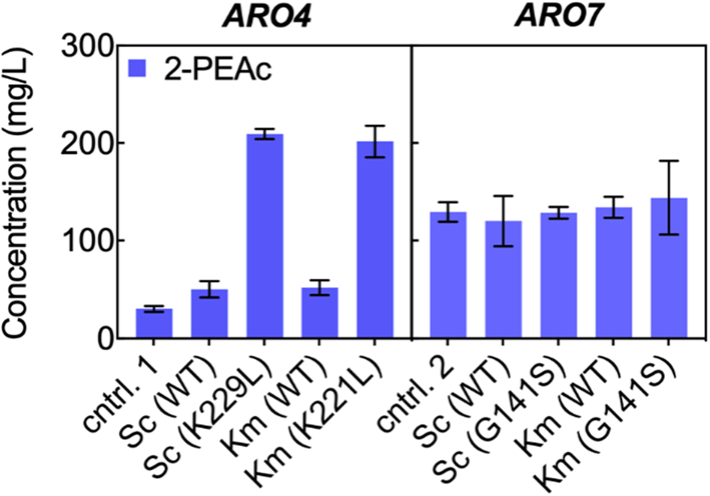
Shikimate pathway enzyme screening. *ARO4* and *ARO7* variants were overexpressed from plasmids with *S. cerevisiae TDH3* promoter (P_*ScTDH3*_) and were screened for 2-phenylethyl acetate (2-PEAc) biosynthesis. 2-PEAc was quantified by GC-FID after 18 h cultivation at 30 °C from an initial OD_600_ of 0.05 in 25 mL SD-H medium. The first control strain (cntrl.1) was *K. marxianus* CBS 6556 *ura3Δ his3Δ* harboring a low-copy number empty vector. The second control strain (cntrl.2) is *K. marxianus* CBS 6556 *ura3Δ his3Δ abz1*::(P_*ScTDH3*_)*KmARO4*^*K221L*^- (P_*ScTDH3*_)*KmARO10* harboring the empty vector. All experiments were performed in biological triplicates. Bars represent the mean, while error bars represent the standard deviation

It has been shown that *S. cerevisiae ARO4* and *ARO7* are feedback inhibited by tyrosine, thus limiting Shikimate pathway flux. The inhibition effect is eliminated in the feedback insensitive variants *ScARO4*^*K229L*^ and *ScARO7*^*G141S*^ [10]. To assess potential feedback inhibition in *K. marxianus*, we overexpressed a series of wild type and mutant *ARO4* and *ARO7* genes from both *S. cerevisiae* BY4742 and *K. marxianus* CBS 6556. In a synthetic defined medium, *K. marxianus* CBS 6556 *ura3Δ his3Δ* accumulated more ester (2-PEAc) than alcohol (2-PE; Additional file 1: Figure S2). As such, for all plasmid-based overexpression experiments we used 2-PEAc titer to quantify the effect of mutations found in *S. cerevisiae* on the corresponding *K. marxianus* variants. 2-PEAc concentration reached a maximum after 18 h of cultivation in the selective medium at 30 °C, this time point was consequently used for enzyme screening. A K229L mutation in *ScARO4* alleviated the inhibition effect and resulted in an increase in 2-PEAc biosynthesis from 31 ± 7 to 209 ± 5 mg/L when overexpressed in *K. marxianus* CBS 6556 *ura3Δ his3Δ* (Fig. 3). The *ScARO4* mutation mapped to K221L in *KmARO4*, overexpression of which also produced an increased amount of 2-PEAc (202 ± 16 mg/L). Feedback inhibition alleviation of *ARO7* was tested with *ScARO7*^*G141S*^ and *KmARO7*^*G141S*^; however, no increase in 2-PEAc production was observed. Given that there was no difference in 2-PEAc biosynthesis between the overexpression of *K. marxianus* and *S. cerevisiae* homologs of *ARO4*, *ARO7*, and their corresponding mutants, we decided to use the regulation-insensitive *K. marxianus* variants, *KmARO4*^*K221L*^ and *KmARO7*^*G141S*^, in the refactoring experiments along with wild-type *KmPHA2*.

### Validation of pathway refactoring promoters

**Fig. 4:**
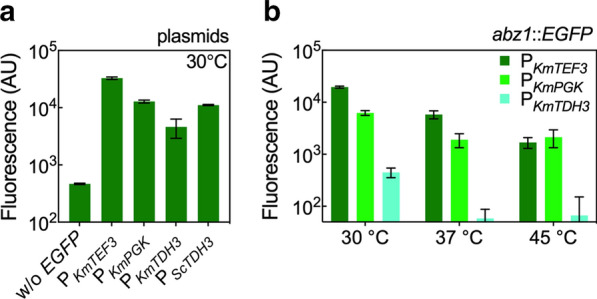
Promoters to construct the Shikimate pathway refactoring library. **a** Transcriptional strengths of constitutive promoters from *K. marxianus* and *S. cerevisiae* were quantified by overexpression of *EGFP* on a low copy-number plasmid. Three characterized promoters from *K. marxianus* CBS 6556 (P_*KmTEF3*_, P_*KmPGK*_, and P_*KmTDH3*_) were used to construct the Shikimate pathway library. **b** The temperature effect on chromosomal expression of *EGFP* from variable strength promoters. Expression cassettes of *EGFP* driven by P_*KmTEF3*_, P_*KmPGK*_ and P_*KmTDH3*_ were integrated into the genome of *K. marxianus* CBS 6556 *ura3Δ his3Δ* at the *ABZ1* locus. All fluorescence measurements were taken using flow cytometry. All experiments were performed in biological triplicates. Bars represent the mean of background-subtracted fluorescence, while error bars represent the standard deviation

Pathway refactoring to balance and increase pathway flux requires promoters of variable strengths; as such, we selected three previously characterized promoters with high, medium, and low expression [40]. Promoters were cloned from the 700 bp upstream of start codons (ATG) of native *K. marxianus TEF3*, *PGK*, and *TDH3* genes to produce P_*KmTEF3*_, P_*KmPGK*_, and P_*KmTDH3*_. Figure 4a shows the resulting *EGFP* fluorescence as measured by flow cytometry from plasmid overexpression at 30 °C. P_*KmTDH3*_ produced the lowest fluorescence, P_*KmPGK*_ exhibited a 2.6-fold increase in *EGFP* expression, and P_*KmTEF3*_ produced the highest expression, 7.8-fold above that produced from P_*KmTDH3*_. A negative control without *EGFP* expression demonstrated a low fluorescence background, while a positive control with *EGFP* constitutively expressed from P_*ScTDH3*_ is provided for comparison with a known high-level *S. cerevisiae* promoter that is functional in *K. marxianus* [44].

Preliminary Shikimate pathway refactoring experiments suggested that 2-PEAc formation was repressed in *K. marxianus* CBS 6556 *ura3Δ his3Δ* when cultured in rich medium (YPD; Additional file 1: Figure S2). In addition, plasmid expression significantly reduced growth rate at elevated temperatures (Additional file 1: Figure S3). For these reasons we conducted all refactoring experiments for 2-PE biosynthesis in YPD medium, thus necessitating chromosomal gene integration. Importantly, the relative expression from the promoter set was maintained after integration (Fig. 4b). With respect to evaluating promoters at higher temperatures, *EGFP* was found to be insensitive to temperature between 30 to 45 °C and served as a reliable expression reporter for temperature effects (Additional file 1: Figure S4). At 37 °C, the strengths of P_*KmTEF3*_ and P_*KmPGK*_ decreased by ~ 70%, while P_*KmTDH3*_ decreased expression by 88% from that observed at 30 °C. Heterologous gene expression driven by P_*KmTEF3*_ was further reduced at 45 °C, exhibiting only 10% of that at 30 °C. The P_*KmPGK*_ and P_*KmTDH3*_ expressions at 45 °C were comparable to those at 37 °C. Despite decreased expression at elevated temperatures, the promoter set provided a substantial range at 30 and 37 °C. A reasonable range in promoter expression was also maintained at 45 °C, but the effect was limited to differences between P_*KmPGK*_ and P_*KmTDH3*_, as P_*KmPGK*_ and P_*KmTEF3*_ produced similar expression levels.

### Shikimate pathway refactoring in *K. marxianus*

**Fig. 5:**
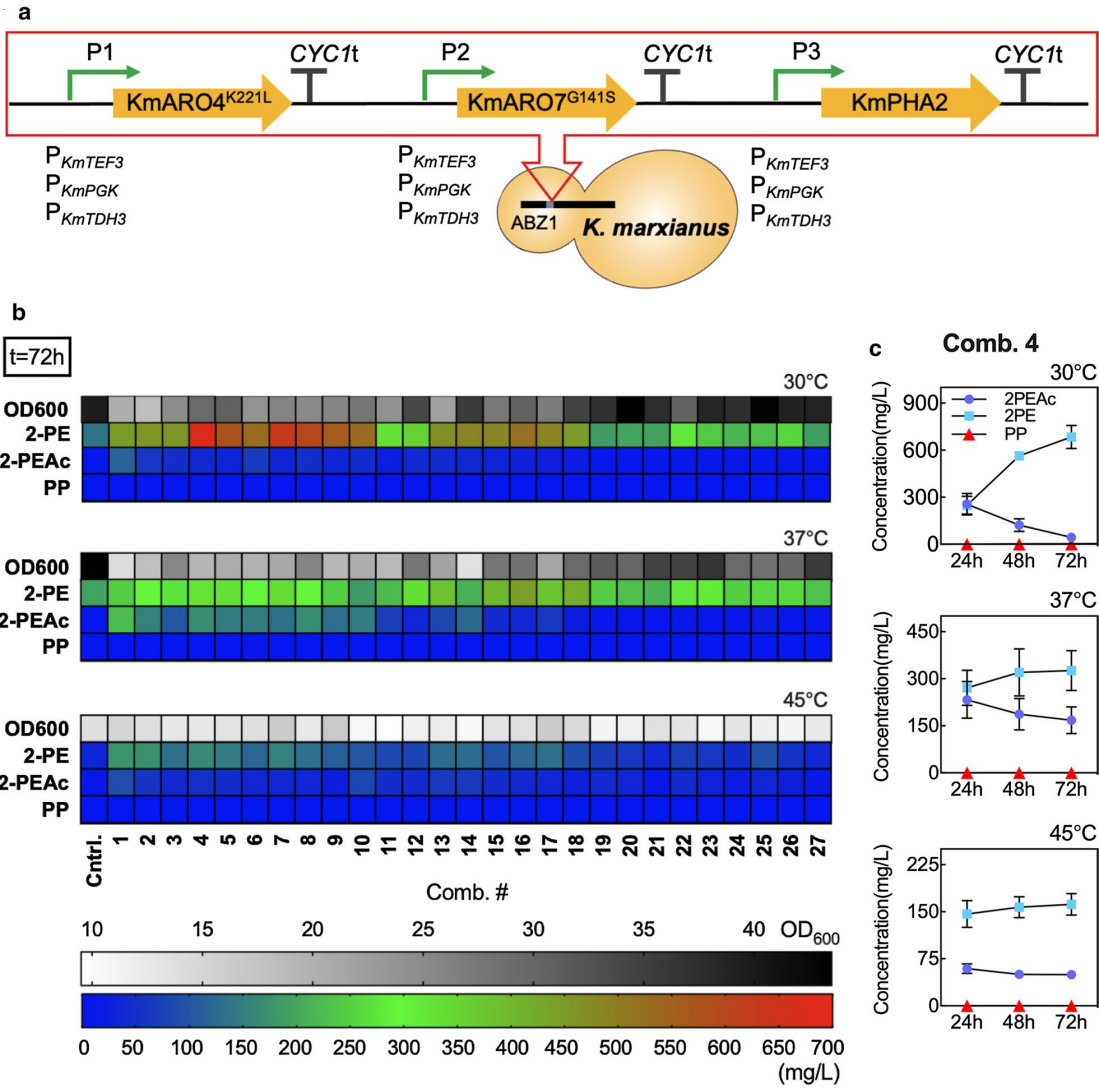
Shikimate pathway refactoring for enhanced 2-PE biosynthesis. **a** Schematic representation of pathway constructs for the library consisting of all 27 combinations of P_*KmTEF3*_, P_*KmPGK*_, and P_*KmTDH3*_ promoters for expression of *KmARO4*^*K221L*^, *KmARO7*^*G141S*^, and *KmPHA2*. **b** OD_600_, extracellular accumulation of 2-phenylethanol (2-PE), 2-phenylethyl acetate (2-PEAc) and phenylpyruvate (PP) in all refactored pathway combinations after 72 h of cultivation at 30, 37 and 45 °C in 25 mL YPD medium. The negative control strain (Cntrl.) is *K. marxianus* CBS 6556 *ura3Δ his3Δ*. **c** Extracellular formation of 2-PE, 2-PEAc and PP over time and at different temperatures for *K. marxianus* CBS 6556 *ura3Δ his3Δ* with refactored Shikimate pathway combination 4 (Comb.4). All experiments were performed in biological triplicates. Bars represent the mean, while error bars represent the standard deviation

Given a validated promoter set with variable expression, we set out to create a series of *K. marxianus* strains with a refactored Shikimate pathway. The library included 27 unique combinations of variable expressions of *KmARO4*^*K221L*^, *KmARO7*^*G141S*^, and *KmPHA2*. As a first step, integration plasmids targeting the *ABZ1* locus with a three-gene insert were constructed. Expression cassettes and inserts are depicted in Fig. 5a. All plasmids and resulting integrated strains were verified by multiple Sanger sequencing experiments. Of note, the refactored pathway was integrated into the genome leaving intact the native Shikimate pathway. This was done so as to not disrupt native Shikimate pathway functions and because the wild type pathway already produces upward of ~ 150 mg/L of 2-PE, thus providing a strong starting point for pathway engineering.

Figure 5b presents the OD_600_ along with the titer of 2-PE, 2-PEAc, and PP produced by each refactored pathway after 72 h of culture at 30, 37, and 45 °C. This time point was used for pathway screening because all strains, including those with a refactored pathway as well as the base strain CBS6556 *ura3Δ his3Δ*, showed the highest extracellular concentration of 2-PE after 72 h cultivation with 20 g/L D-glucose (Additional file 1: Figure S5). The same trend was not observed for 2-PEAc formation, which decreased by 81 ± 11% on average from 24 to 72 h at 30 °C. The concentration of PP was found to be nearly undetectable and unchanging throughout the course of experiments at all temperatures.

Multilinear regression of 2-PE concentration resulting from the refactored pathway combinations at 30 °C revealed statistically significant effects from the overexpression of each gene. The magnitude of the *KmARO4*^*K221L*^ effect was the largest, 4.0- and 3.7-fold greater than that of *KmARO7*^*G141S*^ and *KmPHA2* overexpression. Interaction effects between genes were not found to be significant. Post-hoc analysis by multiple t tests showed that pathway combination 4 (Comb.4) with P_*KmTEF3*_ driving expression of *KmARO4*^*K221L*^ and *KmPHA2*, and P_*KmPGK*_ for overexpression of *KmARO7*^*G141S*^ resulted in the highest 2-PE titer, 684 ± 73 mg/L (Fig. 5b, direct comparison shown in Additional file 1: Figure S6). The large effect of *KmARO4*^*K221L*^ overexpression was observable in the combinations which have a high or medium expression of this gene. At 30 °C, Comb.1–9 and 10–18 produced on average 563 ± 121 and 493 ± 88 mg/L of 2-PE, respectively, while Comb.19–27 only produced 257 ± 45 mg/L of 2-PE (Additional file 1: Figure S7).

Analysis of the data collected at 30 °C also revealed another trend, that of decreased biomass accumulation with increased 2-PE production. Correlation analysis between specific 2-PE titer (mg L^−1^ OD^−1^) and OD_600_ after 72 h of cultivation resulted in a Pearson coefficient, *r*, of −0.89 (Additional file 1: Figure S8). A similar correlation with a Pearson coefficient, *r*, of − 0.74 was also found between total 2-PE titer (mg/L) and OD_600_.

Given that *K. marxianus* CBS 6556 *ura3Δ his3Δ* exhibited comparable growth rate at 30 and 45 °C (Additional file 1: Figure S3), we used the refactored pathway library to investigate any potential temperature effects on both biomass accumulation and 2-PE biosynthesis. Figure 5b and Additional file 1: Figure S7 show that OD_600_ of the negative control strain at 45 °C was only half of that observed at 30 and 37 °C. 2-PE production, both in terms of titer (mg/L) and specific productivity (mg L^−1^ OD^−1^), was highest at 30 °C and decreased as temperature increased to 37 and 45 °C (Fig. 5b, Additional file 1: Figures S5, and S9). For example, 2-PE production from Comb.4 at 72 h decreased from a high of 23 ± 2 mg L^−1^ OD^−1^ at 30 °C to 17 ± 3 mg L^−1^ OD^−1^ at 37 °C and 9 ± 1 mg L^−1^ OD^−1^ at 45 °C (Additional file 1: Figure S9). The time course production of the three relevant metabolites (2-PEAc, 2-PE, and PP) at 30, 37, and 45 °C for the highest producing strain also demonstrated that 30 °C was the optimal temperature to minimize the acetylation of 2-PE (Fig. 5c). As such, we only considered 30 °C cultures for additional analysis and subsequent mutations of the engineered strain with Comb.4 integrated into the genome to enhance 2-PE biosynthesis.

### Ehrlich pathway engineering and fed-batch operation

**Fig. 6:**
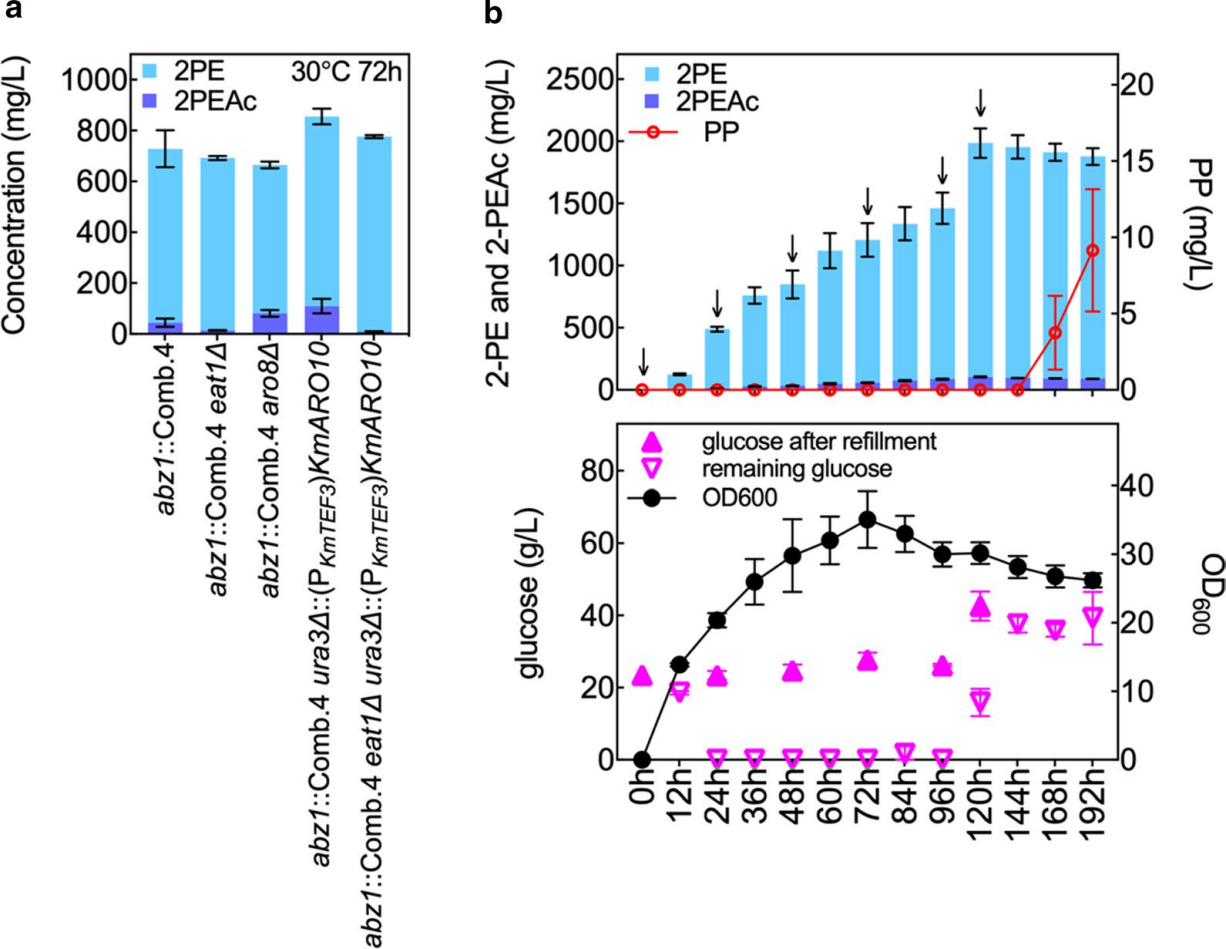
Ehrlich pathway engineering and cultivation optimization. **a** Screening of Ehrlich pathway mutations for enhanced 2-PE biosynthesis. **b** 2-phenylethanol (2-PE), 2-phenylethyl acetate (2-PEAc), phenylpyruvate (PP) and biomass (OD_600_) accumulation from d-glucose in fed-batch operation of the optimal strain, *K. marxianus* CBS 6556 *eat1Δ his3Δ ura3Δ*::(P_*KmTEF3*_)*KmARO10*, *abz1*::(P_*KmTEF3*_)*KmARO4*^*K221L*^-(P_*KmPGK*_)*KmARO7*^*G141S*^-(P_*KmTEF3*_)*KmPHA2*. Arrows indicated glucose addition. All experiments were performed in biological triplicates. Bars represent the mean, while error bars represent the standard deviation

To further increase 2-PE biosynthesis, we explored additional mutations to increase Ehrlich pathway flux and reduce ester production (Fig. 6a). We first disrupted *KmARO8*, knockout of which is known in *S. cerevisiae* to upregulate *ARO10* expression and increase Ehrlich pathway flux [45]. In the engineered strain with integrated Comb.4, loss of *KmARO8* function had little to no effect on 2-PE and 2-PEAc biosynthesis after 72 h of cultivation. Conversely, P_*KmTEF3*_-driven overexpression of *KmARO10* integrated at the *URA3* locus increased 2-PE and 2-PEAc production by 10% and 146% over Comb.4, producing 746 ± 31 and 110 ± 29 mg/L, respectively. Disruption of the ethanol acetyltransferase *KmEAT1* increased the ratio of 2-PE to 2-PEAc by preventing the conversion of alcohol to ester but did not completely eliminate 2-PEAc biosynthesis. The combined effect of *KmARO10* overexpression and *KmEAT1* disruption produced the highest amount of 2-PE, 766 ± 6 mg/L, from 20 g/L d-glucose, with only 10 ± 1 mg/L 2-PEAc at 30 °C after 72 h of cultivation.

During time-course analysis of the pathway refactoring experiments, we observed that D-glucose was depleted by 24 h in the batch culture at 30 °C (Additional file 1: Figure S10). As such, we tested the highest producing strain (*K. marxianus* CBS 6556 *his3Δ eat1Δ, abz1*::(P_*KmTEF3*_)*Km*ARO4^K221L^-(P_*KmPGK*_)*KmARO7*^*G141S*^-(P_*KmTEF3*_)*KmPHA2*, *ura3Δ*::(P_*KmTEF3*_)*KmARO10*) under fed-batch conditions with repeated addition of D-glucose every 24 h, replenishing the culture to 20 g/L (Fig. 6b). 2-PE titer kept increasing until 120 h, reaching a plateau at 1943 ± 63 mg/L. Until 120 h of cultivation, PP remained undetectable and 2-PEAc remained below 110 mg/L. At longer times, 2-PE biosynthesis stalled, glucose consumption decreased, biomass accumulation plateaued, and PP titer increased, suggesting a potential endpoint to the process.

## Discussion

A central challenge in creating new high-producing strains for the industrial bioproduction of chemicals is the rapid creation of new strains with optimized metabolism [30, 46]. One critical component of this is moving beyond plasmid-based expression and creating new strains with integrated, optimized pathways in the genome, thus facilitating translation from laboratory screens to industrial use. In this work, we set out to create a multigene integration tool that facilitates pathway refactoring in the stress-tolerant yeast *K. marxianus*. Our work expands the *K. marxianus* metabolic engineering toolbox, which includes sequential untargeted gene integration using the *URA3* blaster selection cassette [47], random simultaneous integration of multiple genes [48], genome-editing by CRISPR-Cas9 [27, 38, 39], and gene regulation by CRISPRi [8]. Missing from these techniques is the ability to integrate multiple genes into desired sites in the genome at high efficiency. With a CRISPR-Cas9 induced double-stranded break at the target locus, we demonstrated that up to three unique expression cassettes concatenated into a single insert can be integrated with an efficiency that is comparable to single-gene integration. Efficiency was observed to be as high as 95% for a single gene integration into the *URA3* locus. The integration of three genes into the *ABZ1* locus using a single genetic insert 4994 bp in length occurred at an efficiency of 58.3%, statistically similar to the efficiency of integrating a single-gene insert (1730 bp) and a two-gene insert (3343 bp) at the same site (Fig. 2b). Moreover, the CRISPR-mediated system developed here shortens the experimental time required to create multigene insertions. A triple-gene integration into a targeted genomic site can be accomplished within 4 days from transformation to confirmation (Additional file 1: Figure S11). Given a promoter set with a wide transcriptional range, we used this new genomic integration tool to create the full factorial library of three Shikimate pathway genes *ARO4*, *ARO7*, and *PHA2* at three different expression levels (Figs. 4 and 5).

2-PE is a valuable compound for a variety of different applications including next-generation biofuels and flavor and fragrance additives for food, beverages, perfumes, and personal care products. As a potential alternative to chemical synthesis and isolation from natural sources, bioproduction from engineered yeast and bacteria has been investigated by a number of researchers [17–19, 49]. One of these works was able to increase 2-PE production in *K. marxianus* to upward of 1.1 g/L by growth selection in the presence of a toxic l-phenylalanine paralog, *p*-fluorophenylalanine [18]. The concept of the screen was to identify strains that overproduce phenylalanine, a major product of the Shikimate pathway and a precursor to 2-PE via the Ehrlich pathway. While mutations to the high producing strain were not identified in the growth screen, the study suggested that rational engineering to increase flux along the Shikimate pathway should prove valuable. Our Shikimate pathway refactoring results support this conclusion: increased flux to 2-PE was achieved by increasing and balancing the expression of mutant variants of *Km*ARO4, *Km*ARO7, and wild type *Km*PHA2 (Fig. 5).

Previous work in *S. cerevisiae* suggested that *ARO4* and *ARO7* are negatively regulated by tyrosine, thus reducing flux through the Shikimate pathway [10, 50–52]. A K229L mutation alleviated this feed-back inhibition in *ScARO4* as evidenced by an ~ 80-fold increase in 2-PE, reaching a titer of 32 mg/L with glucose as a feedstock [10]. A G141S or T226I mutation to *ScARO7* also relieved the suppression induced by tyrosine [51, 52]. Co-overexpressing *ScARO7*^G141S^ and *ScARO4*^K221L^ increased flux along the Shikimate pathway and boosted 2-PE formation by 200-fold, from 0.4 to ~ 80 mg/L [10]. In the *K. marxianus* homologs, the K229L mutation in *ScARO4* maps to *KmARO4*^K221L^, while the *ScARO7* mutation occurs at the same position in the *K. marxianus* variant, G141S. Plasmid-based overexpression of the putatively feedback-insensitive *KmARO4*^K221L^ in *K. maxianus* CBS 6556 *ura3Δ his3Δ* increased the 2-PEAc titer from 31 ± 7 to 202 ± 16 mg/L, a nearly sevenfold increase (Fig. 3). The overexpression of *KmARO7*^G141S^ in *K. marxianus* CBS 6556 *ura3Δ his3Δ abz1*::(P_*ScTDH3*_)*KmARO4*^K221L^- (P_*ScTDH3*_)*KmARO10* did not further enhance the 2-PEAc biosynthesis (Fig. 3). Overexpressing the *S. cerevisiae* variants in *K. marxianu*s produced similar results, that is, alleviating *ARO4* inhibition was beneficial but overexpression of the *ARO7* mutant did not increase pathway flux. Taken together, these results suggest key differences between *K. marxianus* and *S. cerevisiae* in Shikimate pathway metabolism: (1) *KmARO4* still suffers from but appears to be less sensitive to feedback inhibition; and (2) feedback inhibition suppressing *KmARO7* activity was not observed.

By refactoring the Shikimate pathway, we were able to identify a condition that enhanced 2-PE biosynthesis with high expression of *KmARO4*^K221L^ and *KmPHA2*, and medium expression of *KmARO7*^G141S^. Additional gains in 2-PE biosynthesis were not achieved with higher ARO7 expression potentially due to the interplay between tyrosine feedback inhibition, which was alleviated in *KmARO4 *^*K221L*^ and *KmARO7*^G141S^, and the concentration of tyrosine precursors, specifically the ARO7 product prephenate. The optimized pathway produced 684 ± 73 mg/L of 2-PE after 72 h of shaking at 30 °C in YPD medium, achieving 62% of that in the previously reported toxic phenylalanine paralog screen [18]. This result suggests that while increasing Shikimate pathway flux is important, other mutations might also be beneficial including Ehrlich pathway engineering. Overexpression of the decarboxylation step encoded by *KmARO10* further increased 2-PE titer to 746 ± 31 mg/L, but similar success was not achieved in overexpressing genes encoding putative alcohol dehydrogenases. Specifically, screening various native and heterologous *ADH* variants did not increase 2-PE production (Additional file 1: Figure S12), suggesting that *K. marxianus* CBS 6556 *ura3Δ his3Δ* maintains a high native capacity to reduce the intermediate, phenylacetaldehyde. The terminal Ehrlich pathway step acetylates 2-PE into 2-PEAc primarily via Eat1 activity [8, 53]. With the disruption of *KmEAT1* the extracellular formation of 2-PEAc decreased by 91%, from 110 ± 29 to 10 ± 0.5 mg/L. We previously identified *KmATF1* in *K. marxianus* as an alcohol acetyltransferase that is partially responsible for ethyl acetate production [27]. Promiscuous activity of *KmATF1* towards 2-PE may account for 2-PEAc biosynthesis in the absence of *KmEAT1*. The final strain with optimized Shikimate pathway, *KmARO10* overexpression, and *KmEAT1* disruption produced a high 766 ± 6 mg/L 2-PE titer from 20 g/L of D-glucose in 25 mL YPD medium with shake flask experiments.

*Kluyveromyces marxianus*’ fast growth and thermotolerance to temperature upward of 45–50 °C are valuable traits beneficial to bioprocess design [46]. As such, we investigated the effect of increased culture temperature on 2-PE biosynthesis using the refactored pathway library. Data presented in Fig. 5 demonstrated that at elevated temperature 2-PE production decreased and the effect of pathway refactoring was minimized. The best Shikimate pathway combination (Comb.1: (P_*KmTEF3*_)*KmARO4*^*K221L*^-(P_*KmTEF3*_)*KmARO7*^*G141S*^-(P_*Km TEF3*_)*KmPHA2*) at 45 °C was limited to 177 ± 20 mg/L of 2-PE, 74% lower than the optimal combination (Comb.4: (P_*KmTEF3*_)*KmARO4*^*K221L*^-(P_*KmPGK*_)*KmARO7*^*G141S*^-(P_*Km TEF3*_)*KmPHA2*) at 30 °C (Fig. 5 and Additional file 1: Figure S5). Production was higher at 37 °C: the best combination (Comb. 16: (P_*KmPGK*_)*KmARO4*^*K221L*^-(P_*KmTDH3*_)*KmARO7*^*G141S*^-(P_*KmTEF3*_)*KmPHA2*) produced 444 ± 196 mg/L, a decrease of 35% compared to the Comb.4 at 30 °C (Fig. 5 and Additional file 1: Figure S5).

While we observed a negative correlation between temperature and 2-PE titer in this work, others have reported increased production in metabolically engineered *K. marxianus* with increasing temperature. For example, an engineered pathway for triacetic acid lactone (TAL) in *K. marxianus* CBS 712 produced the highest titer, 1.24 g/L, at 37 °C, ~ twofold more than that at 30 °C [54]. Two key differences are notable between these experiments: (1) our data showed that the expression level of the promoters used to refactor the Shikimate pathway decreased with increasing temperature (Fig. 4), thus resulting in less impact on flux regulation along the Shikimate pathway at elevated temperatures; and, (2) the TAL experiments were conducted with xylose instead of glucose as the carbon source. Previous transcriptional analysis of temperature effects in *K. marxianus* suggested that glycolysis is down-regulated while the pentose phosphate pathway is up-regulated at 45 °C [42, 55]. As for *K. marxianus* CBS 712, the enzymatic activity of xylose reductase and xylitol dehydrogenase was not hampered by temperatures upward to 41 °C [56]. The decrease in glycolytic activity in our experiments was a likely factor in the loss of 2-PE production and is supported by the significant decrease in biomass accumulation observed at 45 °C (Fig. 5b and Additional file 1: Figure S7).

With respect to process design, we further increased 2-PE production through fed-batch operation adding glucose every 24 h over a 120-h experiment (Fig. 6b). Similar to other reports, in our long-term cultures we observed that 2-PE was toxic to *K. marxianus* [14, 57]. This toxicity is observed after 120 h of fed-batch cultivation, when PP began to accumulate and the 2-PE titer plateaued. The effect can be reduced and/or eliminated through in situ removal in a two-phase bioreactor [14]. Such experiments are the focus of on-going process development.

In this work, we focused on refactoring the Shikimate pathway, but other studies have suggested additional metabolic engineering targets that have the potential to make gains in 2-PE production. Two recent studies using *S. cerevisiae* and *Y. lipolytica* as the production host both demonstrated that suppressing the consumption of PEP, one of the substrates of Aro4, to form pyruvate by reducing or even blocking the pyruvate kinase, led to the enhancement of 2-PE biosynthesis [17, 19]. In *Y. lipolytica*, overexpression of the homologous transketolase ylTKT and heterologous phosphoketolases BbxfpK and AcxpkA, which aimed to increase E4P formation from the pentose phosphate pathway, also increased the 2-PE production [19]. However, in *S. cerevisiae*, enhancing the phosphoketolase pathway did not result in higher 2-PE titer. Instead, elimination of the parasitic pathway forming *p*-hydroxyphenylethanol significantly increased the 2-PE production [17].

## Conclusions

The Shikimate pathway can be a productive source of aromatic metabolites useful in various applications, including stilbenoids and flavonoids used for pharmaceuticals and nutraceuticals. Here, we created a library of strains with refactored Shikimate pathway to explore the overproduction of 2-PE, but the same library could also be used to optimize the production of other aromatics. In addition, the multigene integration strategy created here expands the metabolic engineering toolbox for *K. marxianus*, a stress-tolerant yeast that is a promising host for the industrial production of biochemicals.

## Methods

### Molecular cloning and reagents

All molecular cloning reagents and enzymes were purchased from NEB. Plasmids created and used in this work are shown in Additional file 1: Table S2. Plasmids were constructed using the following molecular biology reagents: Q5® High-Fidelity DNA polymerase was used for DNA amplification, NEBuilder® HiFi DNA Assembly Master Mix was used for plasmid assembly, and selected endonucleases were used for plasmid digestion as described in subsequent sections. Primers for gene amplification and mutation were identified in the *K. marxianus* CBS 6556 strain by translated BLAST (tblastn) of annotated genes in the *K. marxianus* DMKU3-1042 or *Saccharomyces cerevisiae* S288C. All primers were purchased from IDT™. All molecular cloning was accomplished in TOP 10 competent *E. coli* (Thermo Fisher Scientific).

All other reagents used in this work were purchased from Fisher Scientific, Sigma-Aldrich or as otherwise noted: BD Difco™ Yeast Nitrogen Base without Amino Acids (Fisher Scientific), CSM-His powder (Sunrise Science Products), CSM-His-Ura powder (Sunrise Science Products), Yeast Synthetic Drop-out Medium Supplements without uracil (Sigma-Aldrich), D-glucose (Fisher Scientific), Gibco™ Bacto™ Yeast Extract (Fisher Scientific), Gibco™ Bacto™ Peptone (Fisher Scientific), 5-FOA (Fisher Scientific), Miller’s LB powder (Sigma-Aldrich), Ampicillin sodium salt (Fisher Scientific), Pfaltz & Bauer Polyethylene glycol 3350 (Fisher Scientific), Lithium acetate dihydrate (Sigma-Aldrich), EDTA disodium salt dihydrate (Sigma-Aldrich), Dithiothreitol (Fisher Scientific), R&D Systems™ Salmon Sperm DNA (Fisher Scientific), 2-Phenylethanol (99.0% min by GC, Millipore Sigma), 2-phenylethyl acetate (99.0% min by GC, Millipore Sigma), Phenylpyruvic acid (97.0% min by HPLC, Millipore Sigma), and Cyclohexane (99.0% min by GC, Fisher Scientific).

### *K. marxianus* strains, media, and cultivation

*Kluyveromyces marxianus* CBS 6556 *ura3Δ his3Δ* was used as a starting strain for all experiments described in this work. All constructed strains are listed in Table S2. Synthetic defined (SD) media was used for all plasmid-based expression experiments. The SD-U medium is defined as 6.7 g/L BD Difco™ Yeast Nitrogen Base without amino acids, 1.92 g/L Yeast Synthetic Drop-out Medium Supplements without uracil, and 20 g/L d-glucose. SD-H and SD-H-U are similar defined but with 0.75 g/L of CSM-His and CSM-His-Ura, respectively. For all pathway refactoring experiments and 2-PE biosynthesis analysis, *K. marxianus* strains were cultivated rich YPD medium (YPD: 10 g/L Gibco™ Bacto™ Yeast Extract, 20 g/L Gibco™ Bacto™ Peptone, 20 g/L d-glucose). 20 g/L agar was added to make solid agar plates. All yeast cultures were conducted in 250 mL baffled shake flasks containing 25 mL of appropriate media. Culturing was conducted in an INFORS HT Multitron incubation shaker with temperature control set to 30, 37 and 45 °C as needed.

### CRISPR-Cas9 gene disruption and integration plasmids

CRISPR plasmids (pCRISPR) were constructed using pIW601 (Addgene ID 98907) linearized at PspXI and re-assembled with a 60 bp insert containing 20 bp upstream and downstream homology as well as the 20 bp target sequence by Gibson assembly. Each insert was constructed by annealing the two complementary 60 bp primers. For each locus, five crRNA sequence candidates were chosen based on the on-target cutting efficiency using the sgRNA design tool hosted by Broad Institute (https://portals.broadinstitute.org/gpp/public/analysis-tools/sgrna-design) [58] and the uniqueness across the *K. marxianus* genome using a CRISPR/Cas9 target online predictor, CCTop (https://crispr.cos.uni-heidelberg.de/) [59].

Homology donor plasmids (pHD) for gene integration were constructed from pIW578. pIW578 was digested with two restriction enzymes, NotI and EagI, to sequentially insert 700 bp up- and down-stream homology of the 20 bp crRNA targeting locus on the *K. marxianus* CBS 6556 *ura3Δ his3Δ* genome. The homology donor vectors were then digested at XmaI and XhoI to insert gene expression cassette(s) for targeted integration. For example, pHDs with single- (*EGFP*), dual- (*DSRED* and *EGFP*), and triple-gene (*DSRED*, *YFP*, and *EGFP*) cassettes were created as listed in Table S2.

### Enzyme screening and Shikimate pathway refactoring plasmids

All plasmids used for enzyme screening were constructed by replacing EGFP in pIW1135 with the new gene (Table S2). As for the expression plasmids for single-amino acid mutated variants of *ScARO4*, *ScARO7*, *KmARO4*, and *KmARO7*, a 53-bp insert containing the mutation flanked by 25 bp up- and down-stream homology of the to-be-replaced triplet of bases was designed for each gene. The vector expressing the corresponding native protein was linearized by a pair of primers designed to eliminate the exact codon to be substituted.

Refactored Shikimate pathways were integrated into *K. marxianus* CBS6556 *ura3Δ his3Δ* in a three-step process. First, variable strength promoters were validated, second, plasmids with multiple gene expression cassettes were constructed, and finally, the 27 multigene expression combinations were integrated into the *ABZ1* locus. Briefly, promoter validation plasmids were constructed by replacing the P_*ScTDH3*_ used to drive EGFP expression in pIW1135 with P_*KmTEF3*_, P_*KmPGK*_, and P_*KmTDH3*_ [40] by Gibson assembly. Subsequently, nine expression cassettes using each of the promoters separately for *KmARO4*^*K221L*^, *KmARO7*^*G141S*^ and *KmPHA2* were constructed by replacing the EGFP gene. These expression cassettes were then concatenated into multigene inserts by designing primers with overlapping ends for Gibson assembly. The result was 27 unique integration plasmids with 700 bp up and down-stream homology to *ABZ1* flanking a three-gene overexpression insert. Integration was accomplished as described below.

### *K. marxianus* transformation

*Kluyveromyces marxianus* transformations were accomplished using a modified protocol described in [27]. Briefly, the desired starting *K. marxianus* strain was cultivated in 2 mL YPD for 16 h at 30 °C. 1 mL of the cell culture was harvested by centrifugation at 5000*g* for 1 min in a sterile 1.5 mL Eppendorf tube. After washing twice with 1 mL of sterile ddH_2_O, cells were mixed with 10 µL of 10 mg/mL carrier DNA (R&D Systems™ salmon sperm DNA) by gently vortexing. For gene integration, 1 µg pCRISPR and 0.8 µg pHD were then added. For CRISPR-mediated gene disruption or plasmid-based gene expression, 1 µg pCRISPR or 0.8 µg overexpression plasmid was used. After adding 500 µL of transformation buffer, all components in the tube were mixed thoroughly by pipetting and were incubated at room temperature for 15 min. Subsequently, the mixture was heat shocked at 47 °C for 5 min using a solid heat block containing diH_2_O in each well. Cells were then harvested by centrifugation at 5000*g* for 30 s. Supernatant was removed and cell pellets were resuspended in 500 µL selective media based on the selection marker of the transformed plasmid(s). The transformation buffer contains 40% w/w autoclave sterilized polyethylene glycol 3350 (PEG), 0.45-µm filtered 0.1 M lithium acetate (LiAc), 10 mM Tris–HCl (pH 7.5) with 1 mM EDTA (1 × TE buffer), and 10 mM DTT. Transformants were selected by inoculation in to the appropriate selective liquid medium or by plating directly on selective solid agar medium as described in the following section.

### Strain selection and validation

For gene integration, 50 µL of transformed cell resuspension was first cultivated in 2 mL SD-H-U medium at 37 °C for 36 h. To enrich colonies with expected chromosomal insertion, cells were reinoculated in fresh medium overnight at 30 °C, and subsequently plated on YPD for single colonies (see Figure S11). The efficiency of genomic integration was determined after 16 h incubation on YPD plates at 37 °C. In each case, 28 colonies were randomly picked and screened by a three-primer colony PCR and subsequent gel electrophoresis. Two of the designed primers bind to the genome outside the homology arms (one upstream and one downstream). The third primer binds to the integration cassette (see Fig. 2a). Positive integrations were confirmed with amplicons produced from the upstream primer that binds to the insert and downstream genomic primer. Positive integration amplicons vary in size; the binding location of the upstream genomic primer was adjusted on purpose to maintain a band size difference between positive and negative results. For single-gene integration, in the absence of integration, amplification occurred with the two genomic primers to produce a 1640 bp amplicon, while successful gene integrations produced an 831 bp amplicon. Positive multigene integration produced a ~ 1110 bp amplicon, while the negative result produced a 2023 bp band. Plasmids were removed from selected colonies that gave positive results in the colony PCR analysis by culturing in 1 mL YPD containing 1 g/L 5-Fluoroorotic acid (5-FOA) at 30 °C for 16 h. Single colonies were isolated by plating again on solid YPD. The inserted gene expression cassette(s) were confirmed by Sanger sequencing after genome isolation (YeaStar™ Genomic DNA Kit), fragment amplification, and purification (DNA Clean & Concentrator®-5). The colony selection for plasmid-based gene overexpression was achieved by the outgrowth of the transformed cells in the appropriate selective liquid medium for two times to enrich the number of cells harboring the expected plasmid and then plated on selective agar plates for single colonies ready for use.

To confirm gene disruptions, 50 µL of transformed cell resuspension was inoculated in 2 mL SD-U. After reaching confluency at 37 °C (usually after 24 h cultivation), cells were reinoculated in another 1 mL SD-U for outgrowth at 30 °C overnight and appropriately diluted cell cultures were plated on SD-U to obtain single colonies. After 16 h of growth at 37 °C, 14 randomly picked colonies were screened by amplifying a ~ 500 bp fragment containing the CRISPR-Cas9 targeted site by colony PCR. PCR amplicons were isolated by gel electrophoresis and were sent for Sanger sequencing after column-purification (Zymoclean™ Gel DNA Recovery Kit).

### Microtiter plate measurements

A BioTek®Synergy™ Neo2 multi-mode microplate reader was used to measure the relative fluorescence intensity of yeast cells. Optimization studies revealed that maximum fluorescence was observed when cells reached late exponential phase, approximately 14 h of growth at 30 °C in YPD medium. Due to differences in quantum yield and absolute fluorescence produced from EGFP, YFP, and DSRED, fluorescence measurements varied between samples expressing different fluorescent proteins. 500 µL of cell culture was collected, pelletized, and washed twice with PBS. For EGFP, the washed cell pellets were resuspended in 500 µL of PBS and diluted tenfold prior to measurement (Ex/Em 488/511). For YFP, cell pellets were resuspended in 500 µL of PBS and measured directly (Ex/Em 520/540). Finally, for DSRED, 100 µL PBS was used to resuspend harvested cells before fluorescence quantification (Ex/Em 555/604). All measurements were conducted on 100 µL of resuspended cells in a black Corning™ 96-well flat-bottom microplate (Fischer Scientific).

### Flow cytometry

Single-cell fluorescence quantification was conducted using a BD Accuri™ C6 Plus flow cytometer. Briefly, 500 µL of cell culture was transferred into a 1.5 mL tube and centrifuged at 5000*g* for 1 min. Supernatants were discarded and cell pellets were washed twice with 1 mL PBS. Cell pellets were then resuspended in 500 µL sterile ddH_2_O. Two-microliter of the cell solutions was further diluted in 100 µL ddH_2_O and placed into one well of a clear Corning™ 96-well flat-bottom polystyrene microtiter plate for auto-loading. Standard manufacturer settings of laser, filter, and detector to quantify EGFP fluorescence were used (Ex/Em 488/533). Each run was limited by collecting 10,000 events, and the fluidics rate was 14 µL/min. Histograms of flow cytometry events for data presented in Fig. 4 are shown in Figures S14-S17. For strains with plasmid-based EGFP overexpression cultured in selective medium, samples were taken after 16 h of cultivation at 30 °C. For chromosomal expression in the rich YPD medium, the sampling time for 30 and 37 °C was 14 h, while for 45 °C, samples were analyzed at 10 h.

### Gas chromatography

Analysis of 2-phenylethanol (2-PE), 2-phenylethyl acetate (2-PEAc), and phenylpyruvate (PP) was carried out on a Shimadzu GC-2010 Plus equipped with a Shimadzu AOC-20s autosampler and a Shimadzu AOC-20i auto-injector. The GC suite was coupled to an flame ionization detector (FID). Compounds were separated on an Agilent J&W DB-WAX Ultra Inert column (length: 30 m; inner diameter: 0.32 mm; film thickness: 0.5 µm), using a 21 min temperature program as follows: start temperature of 100 °C, 20 °C/min to 140 °C, 10 °C/min to 150 °C, 5 °C/min to 160 °C, hold for 2 min then increase by 1 °C/min to 170 °C, hold for 2 min, and finally 25 °C/min to 220 °C. Helium was used as the carrier gas at a flow rate of 1.9 mL/min. The sample injection volume was 1 µL. Split mode was used for injection and the ratio was 20:1. The following retention times were determined using standard samples: PP, 4.5 min; 2-PEAc, 8.1 min; 2-PE, 10.1 min.

For sample preparation, 700 µL cell culture was centrifuged for 1 min at 5000*g*, 500 µL supernatant was collected and transferred to a clean 1.5 mL tube with an equal volume of cyclohexane. The mixture was vortexed thoroughly for 30 min. After centrifugation at 10,000*g* for 1 min, 100 µL of the organic layer was transferred into a 2 mL clear Agilent GC vial with glass insert. Standard curves depicting the linear correlation between the concentration of three compounds (PP, 2-PEAc, 2-PE) with the area of peaks from FID were obtained to quantify extracellular metabolite accumulation by different strains. A series of YPD solutions of 2-PE, 2-PEAc and PP with known concentrations were made and extracted by cyclohexane following the same procedure which was used to extract these three compounds from the supernatants of cell cultures.

### Reverse transcription quantitative PCR (RT-qPCR)

The relative expression strengths of promoter P_*KmTEF3*_, P_*KmPGK*_ and P_*KmTDH3*_ were quantified by RT-qPCR according to the *KmARO4*^*K221L*^ expression in three *K. marxianus* CBS 6556 strains with engineered Shikimate pathway (see the refactored combination 1, 10, and 19). These strains differ only in the promoter that drives *KmARO4*^*K221L*^ expression. Total RNA was extracted using the YeaStar™ RNA Kit (Zymo Research). 10 µg RNA was DNAse treated (DNAse I, New England Biolabs) in a 100 µL reaction. The mixture was subsequently purified using the RNA Clean & Concentrator™-25 Kit (Zymo Research). 400 ng column-purified RNA was used for a 20 µL reverse transcription reaction (iScript™ Reverse Transcription Supermix for RT-qPCR, Bio-Rad) and 2 µL cDNA synthesis reaction was directly used for SYBR Green qPCR (SsoAdvanced™ Universal SYBR® Green Supermix, Bio-Rad) conducted on the Bio-Rad CFX Connect™. Fold change of the number of *KmARO4*^*K221L*^ transcripts of each engineered strain (with Comb.1, Comb.10, or Comb.19 integrated at locus *ABZ1*) versus the negative control (*K. marxianus* CBS 6556 *ura3Δ his3Δ*) was calculated using transcription level normalized to GAPDH expression (Additional file 1: Figure S13).

### Glucose assay

The concentration of residual glucose was quantified using a commercially available glucose assay kit (GAGO-20; Sigma-Aldrich). 200 µL of the sample was mixed with 400 µL assay reagents as directed. After reaction for 30 min at 37 °C, the reaction was stopped by addition of 400 µL 12 N H_2_SO_4_. 100 µL of the final solution was then transferred into a clear Corning™ 96-well flat-bottom polystyrene microplate and absorbance was determined at 540 nm using BioTek®Synergy™ Neo2 multi-mode microplate Reader. The region where absorbance is proportional to glucose concentration was identified by the calibration curve. A 0.1 g/L glucose solution stock was made by adding 5 µL YPD containing 20 g/L glucose into 995 µL ddH_2_O and mixing thoroughly. A series of dilution were obtained at 0.04, 0.02, 0.01, and 0.005 g/L to plot a standard curve of absorbance at 540 nm and glucose concentration (Additional file 1: Figure S18). For sample preparation, 500 µL cell cultures were centrifuged down at 5000*g* for 1 min and the supernatant was filtered using a Corning® syringe filter with 0.2 µm-pore membrane. 5 µL of the supernatant was diluted in 995 µL ddH_2_O. If the absorbance of this dilution at 540 nm is below 0.046, the concentration of glucose remaining in the media will be less than 0.2 g/L, which in this work, was considered as “glucose depleted”.

### Statistical analysis

Brown-Forsythe and Welch one-way ANOVA tests were used to compare GFP fluorescence from various genomic integration sites. Multiple linear regression was used to analyze the pathway refactoring experiments. Comparison between the 2-PE titers of any two pathway refactoring combinations was accomplished by multiple *t* tests. The Pearson coefficient, r, was used to represent the correlation between biomass accumulation and 2-PE titer or specific 2-PE production. All data plots and statistical analysis were performed using GraphPad Prism 8 software with a *P* value < 0.05 taken as significant.

## Supplementary Information

**Additional file 1. **Additional Figures and Tables.

## Data Availability

All data generated and analyzed during the current study are available from the corresponding author on reasonable request.

## Abbreviations

2-PE: 2-Phenylethanol
CRISPR: Clustered regularly interspaced short palindromic repeats
Cas9: CRISPR-associated protein 9
E-4-P: Erythrose-4-phosphate
PEP: Phosphoenolpyruvate
PP: Phenylpyruvate
Adh: Alcohol dehydrogenase
2-PEAc: 2-Phenylethyl acetate
ATTase or Atf: Alcohol acetyltransferase
PAR: Phenylacetaldehyde reductase
GRAS: Generally regarded as safe
NHEJ: Non-homologous end joining
HR: Homologous recombination
CRISPR a/i: CRISPR activation and interference
G-6-P: Glucose 6-phosphate
DAHP: 3-Deoxy-d-arabionoheptulosonate-7-phosphate
PABA: Para-aminobenzoate
Trp: Tryptophan
Phe: Phenylalanine
ARO3/ARO4: DAHP synthase
ARO7: Chorismate mutase
PHA2: Prephenate dehydratase
ARO8/ARO9: Aromatic aminotransferase
ARO10: Phenylpyruvate decarboxylase
EAT1: Ethanol acetyltransferase
sgRNA: Single guide RNA
EGFP: Enhanced green fluorescence protein
YFP: Yellow fluorescence protein
DSRED: Red fluorescence protein isolated from Discosoma
ANOVA: Analysis of variance
OD: Optical density
TAL: Triacetic acid lactone
BLAST: Basic local alignment search tool
5-FOA: 5-Fluoroorotic acid
EDTA: Ethylenediaminetetraacetic acid disodium salt dihydrate
LB: Luria–Bertani
GC: Gas chromatography
HPLC: High-performance liquid chromatography
SD: Synthetic defined
DTT: Dithiothreitol
PEG: Polyethylene glycol
Tris-HCl: Tris(hydroxymethyl)aminomethane hydrochloride
TE: Tris-EDTA
Ex/Em: Excitation/emission wavelength in nm
FID: Flame ionization detector
RT-qPCR: Reverse transcription quantitative PCR
GAPDH: Glyceraldehyde 3-phosphate dehydrogenase
