# Supplementary material for: CRISPR-mediated multigene integration enables Shikimate pathway refactoring for enhanced 2-phenylethanol biosynthesis in Kluyveromyces marxianus
Source: Biotechnol Biofuels. 2021 Jan 6;14:3. doi: 10.1186/s13068-020-01852-3 (PMC7788952; doi:10.1186/s13068-020-01852-3)
Supplement: Supplementary file 1 — Additional file 1. Additional Figures and Tables. [file 13068_2020_1852_MOESM1_ESM.docx]

Supplementary Material

**CRISPR-mediated multigene integration enables Shikimate pathway refactoring for enhanced 2-phenylethanol biosynthesis in *Kluyveromyces marxianus***

Mengwan Li^1^, Xuye Lang^1^, Marcos Moran Cabrera^1^, Sawyer De Keyser^1^, Xiyan Sun^1^, Nancy DaSilva^2^, and Ian Wheeldon^1,3^*

1. Department of Chemical and Environmental Engineering, University of California Riverside, Riverside, CA 92521
2. Department of Chemical Engineering, University of California Irvine, Irvine, CA 92697
3. Center for Industrial Biotechnology, University of California Riverside, Riverside, CA 92527

* Corresponding Author. Email: iwheeldon@engr.ucr.edu

Table S1: Elected integration loci on the genome of *K. marxianus* CBS 6556 *ura3Δ his3Δ*

Table S2: Plasmids and strains used in this study

Table S3: Primers used for cloning in this study

Table S4: Sequence of the codon-optimized *rosePAR*

Figure S1: Homology donor length effects on the CRISPR-mediated gene integration in *K. marxianus* CBS 6556 *ura3Δ his3Δ*

Figure S2: Medium effects on 2-PE acetylation in *K. marxianus* CBS 6556 *ura3Δ his3Δ*

Figure S3: Undesirable effects of plasmid reliance on cell growth at elevated temperature for *K. marxianus* CBS 6556 *ura3Δ his3Δ*

Figure S4: EGFP stability over 40-h incubation at 30, 37, and 45 °C

Figure S5: Extracellular formation of 2-phenylethanol (2-PE), 2-phenylethyl acetate (2-PEAc), and phenylpyruvate (PP) after 24, 48, and 72 h cultivation at (A) 30, (B) 37, and (C) 45 °C

Figure S6: Effects of varied expression combinations of *KmARO4^K221L^*, *KmARO7^G141S^*, and *KmPHA2* on 2-PE accumulation after 72 h cultivation at 30 °C

Figure S7: Temperature effects on 2-PE formation and biomass accumulation

Figure S8: Correlation of the (A) 2-PE concentration and (B) specific 2-PE production with biomass accumulation (shown by OD_600_) after 72-h cultivation at 30 °C

Figure S9: Specific production (mg L^-1^ OD^-1^) of 2-PE, 2-PEAc, and PP after 24, 48 and 72 h cultivation at (A) 30, (B) 37, and (C) 45 °C

Figure S10: Extracellular 2-PE and 2-PEAc formation with glucose consumption in *K. marxianus* CBS 6556 *his3Δ* *eat1Δ* *ura3Δ*::*P_KmTEF3_*(*KmARO10*), *abz1*::(*P_KmTEF3_*)*KmARO4^K221L^*-(*P_KmPGK_*)*KmARO7^G141S^*-(*P_KmTDH3_*)*KmPHA2*

Figure S11: Schematic representation of the standard protocol for CRISPR-Cas9 mediated one-step integration up to three genes through yeast transformation to integration confirmation

Figure S12: Effects of plasmid-based or chromosomal overexpression of homologous and heterologous genes encoding alcohol dehydrogenases (Adh) or phenylacetaldehyde reductases (Par) on 2-PE and 2-PEAc biosynthesis

Figure S13: Relative transcriptional activity of the 700-bp natively derived *KmTEF3*, *KmPGK*, and *KmTDH3* promoter for *KmARO4* (including wild-type *KmARO4* and *KmARO4^K221L^*) expression

Figure S14: Histogram events of EGFP expression driven by P*_KmTEF3_*, P*_KmPGK_*, P*_KmTDH3_*, and P*_ScTDH3_* on plasmid at 30 °C, and corresponding cell size distribution

Figure S15: Histogram events of integrated EGFP expression driven by P*_KmTEF3_*, P*_KmPGK_*, and P*_KmTDH3_* at 30 °C, and corresponding cell size distribution

Figure S16: Histogram events of integrated EGFP expression driven by P*_KmTEF3_*, P*_KmPGK_*, and P*_KmTDH3_* at 37 °C, and corresponding cell size distribution

Figure S17: Histogram events of integrated EGFP expression driven by P*_KmTEF3_*, P*_KmPGK_*, and P*_KmTDH3_* at 45 °C, and corresponding cell size distribution

Figure S18: Standard curve of glucose concentration (g/L) to absorbance at 540 nm

Table S1. Elected integration loci on the genome of *K. marxianus* CBS6556 ura3Δ his3Δ. The annotated function of the gene, gene ID (*K. marxianus* DMKU3-1042), * protein sequence ID (*S. cerevisiae* S288C) and the targeted sequence of the sgRNA are shown.

| Locus | Gene function | Gene ID | sgRNA targeting sequence (5’ to 3’) |
| --- | --- | --- | --- |
| *URA3* | Disrupted in *K. marxianus* CBS 6556 *ura3Δ* *his3Δ* | 34714385 | TTGCCGAGTTATCGTCCAAG |
| *XYL2* | Xylose metabolism | * NP_013171.1 | ACGATCGCCAACCTTGACCA |
| *ABZ1* | para-aminobenzoic acid (PABA) biosynthesis | 34717950 | AGAATCTTGCTGTTCGAAGC |
| *LYS1* | Lysine biosynthesis | 34716354 | AAAGAGATACAGCTTCTGTT |
| *SDL1* | Serine metabolism | 34715098 | ATTTGATAATGCAACCCTCT |
| *ARO8* | Phenylalanine biosynthesis and metabolism | 34714734 | GATCCACGACATGCAATACG |
| *EAT1* | Acetylation | * NP_011529.1 | GAGCCTTCAACCAACAAAAA |

Table S2. Plasmids and strains used in this study

| Plasmids | Description | Reference (Addgene #) |
| --- | --- | --- |
| pIW601 | P*_ScTEF1_*-KmCas9-SV40-*ScCYC1*t, P*_KmPRP1_*-tRNA^Gly^-PspXI recognition site­-SUP4 | Löbs et al.^1^ (98907) |
| pIW578 | Replacing the *URA3* marker in the pJSKM316-GPD with *HIS3* marker | Lee et al.^2^ |
| pIW538 | pIW601with *URA3* targeting sgRNA | This study |
| pIW447 | pIW601with *XYL2* targeting sgRNA | This study |
| pIW1134 | pIW601with *ABZ1* targeting sgRNA | This study |
| pIW1137 | pIW601with *LYS1* targeting sgRNA | This study |
| pIW1132 | pIW601with *SDL1* targeting sgRNA | This study |
| pIW21 (pATF1G) | pET-28(+) derivative with *ScATF1*-*EGFP* insertion | Zhu et al.^3^ |
| pIW1198 | 700bp *URA3* up and downstream, pIW578 with *EGFP* | This study |
| pIW1136 | 700bp *XYL2* up and downstream, pIW578 with *EGFP* | This study |
| pIW1135 | 700bp *ABZ1* up and downstream, pIW578 with *EGFP* | This study |
| pIW1146 | 700bp *LYS1* up and downstream, pIW578 with *EGFP* | This study |
| pIW1149 | 700bp *SDL1* up and downstream, pIW578 with *EGFP* | This study |
| pIW247 | P*_ScPGK1_*-*ATF1*-*DSRED*-*ScTEF2*t, P*_ScTDH2_-ALD6*-*YFP*-Doc(Cp)*-ScHXT7*t | Lin et al.^4^ |
| pIW1189 | Replacing the *EGFP* in pIW1135 with *DSRED* | This study |
| pIW1190 | Replacing the *EGFP* in pIW1135 with *YFP* | This study |
| pIW1147 | pIW1135 with P*_ScTDH3_*-*DSRED*-*ScCYC1*t | This study |
| pIW1148 | pIW1147 with P*_ScTDH3_*-*YFP*-*ScCYC1*t | This study |
| pIW1199 | Replacing the *EGFP* in pIW1135 with *ScARO4* | This study |
| pIW1200 | Replacing the *EGFP* in pIW1135 with *ScARO7* | This study |
| pIW1201 | Replacing the *EGFP* in pIW1135 with *KmARO4* | This study |
| pIW1202 | Replacing the *EGFP* in pIW1135 with *KmARO7* | This study |
| pIW1203 | Replacing the *EGFP* in pIW1135 with *ScARO4^K229L^* | This study |
| pIW1204 | Replacing the *EGFP* in pIW1135 with *ScARO7^G141S^* | This study |
| pIW1205 | Replacing the *EGFP* in pIW1135 with *KmARO4^K221L^* | This study |
| pIW1206 | Replacing the *EGFP* in pIW1135 with *KmARO7^G141S^* | This study |
| pIW1222 | pIW1205 with P*_ScTDH3_*-*KmARO10*-*ScCYC1*t | This study |
| pIW1246 | Replacing the P*_ScTDH3_* in pIW1135 with P*_KmTEF3_* | This study |
| pIW1247 | Replacing the P*_ScTDH3_* in pIW1135 with P*_KmPGK_* | This study |
| pIW1248 | Replacing the P*_ScTDH3_* in pIW1135 with P*_KmTDH3_* | This study |
| pIW1234 | A blank vector made from pIW1135 for individual insertion of the 27 refactored overexpression combinations | This study |
| pIW1235 | Replacing the *EGFP* in pIW1246 with *KmARO4^K221L^* | This study |
| pIW1236 | Replacing the *EGFP* in pIW1247 with *KmARO4^K221L^* | This study |
| pIW1237 | Replacing the *EGFP* in pIW1248 with *KmARO4^K221L^* | This study |
| pIW1229 | Replacing the *EGFP* in pIW1246 with *KmARO7^G141S^* | This study |
| pIW1238 | Replacing the *EGFP* in pIW1247 with *KmARO7^G141S^* | This study |
| pIW1239 | Replacing the *EGFP* in pIW1248 with *KmARO7^G141S^* | This study |
| pIW1230 | Replacing the *EGFP* in pIW1246 with *KmPHA2* | This study |
| pIW1240 | Replacing the *EGFP* in pIW1247 with *KmPHA2* | This study |
| pIW1241 | Replacing the *EGFP* in pIW1248 with *KmPHA2* | This study |
| pIW1251 | pIW1234 with P*_KmTEF3_*-*KmARO4^K221L^*-*ScCYC1*t, P*_KmTEF3_*-*KmARO7^G141S^*-*ScCYC1*t, P*_KmTEF3_*-*KmPHA2*-*ScCYC1*t | This study |
| pIW1252 | pIW1234 with P*_KmTEF3_*-*KmARO4^K221L^*-*ScCYC1*t, P*_KmTEF3_*- *KmARO7^G141S^*-*ScCYC1*t, P*_KmPGK_*-*KmPHA2*-*ScCYC1*t | This study |
| pIW1253 | pIW1234 with P*_KmTEF3_*-*KmARO4^K221L^*-*ScCYC1*t, P*_KmTEF3_*-*KmARO7^G141S^*-*ScCYC1*t, P*_KmTDH3_*-*KmPHA2*-*ScCYC1*t | This study |
|  |  |  |
| Plasmids | Description | Reference (Addgene #) |
| pIW1254 | pIW1234 with P*_KmTEF3_*-*KmARO4^K221L^*-*ScCYC1*t, P*_KmPGK_*-*KmARO7^G141S^*-*ScCYC1*t, P*_KmTEF3_*-*KmPHA2*-*ScCYC1*t | This study |
| pIW1255 | pIW1234 with P*_KmTEF3_*-*KmARO4^K221L^*-*ScCYC1*t, P*_KmPGK_*-*KmARO7^G141S^*-*ScCYC1*t, P*_KmPGK_*-*KmPHA2*-*ScCYC1*t | This study |
| pIW1256 | pIW1234 with P*_KmTEF3_*-*KmARO4^K221L^*-*ScCYC1*t, P*_KmPGK_*-*KmARO7^G141S^*-*ScCYC1*t, P*_KmTDH3_*-*KmPHA2*-*ScCYC1*t | This study |
| pIW1257 | pIW1234 with P*_KmTEF3_*-*KmARO4^K221L^*-*ScCYC1*t, P*_KmTDH3_*-*KmARO7^G141S^*-*ScCYC1*t, P*_KmTEF3_*-*KmPHA2*-*ScCYC1*t | This study |
| pIW1258 | pIW1234 with P*_KmTEF3_*-*KmARO4^K221L^*-*ScCYC1*t, P*_KmTDH3_*-*KmARO7^G141S^*-*ScCYC1*t, P*_KmPGK_*-*KmPHA2*-*ScCYC1*t | This study |
| pIW1259 | pIW1234 with P*_KmTEF3_*-*KmARO4^K221L^*-*ScCYC1*t, P*_KmTDH3_*-*KmARO7^G141S^*-*ScCYC1*t, P*_KmTDH3_*-*KmPHA2*-*ScCYC1*t | This study |
| pIW1260 | pIW1234 with P*_KmPGK_*-*KmARO4^K221L^*-*ScCYC1*t, P*_KmTEF3_*-*KmARO7^G141S^*-*ScCYC1*t, P*_KmTEF3_*-*KmPHA2*-*ScCYC1*t | This study |
| pIW1261 | pIW1234 with P*_KmPGK_*-*KmARO4^K221L^*-*ScCYC1*t, P*_KmTEF3_*-*KmARO7^G141S^*-*ScCYC1*t, P*_KmPGK_*-*KmPHA2*- *ScCYC1*t | This study |
| pIW1262 | pIW1234 with P*_KmPGK_*-*KmARO4^K221L^*-*ScCYC1*t, P*_KmTEF3_*-*KmARO7^G141S^*-*ScCYC1*t, P*_KmTDH3_*-*KmPHA2*- *ScCYC1*t | This study |
| pIW1263 | pIW1234 with P*_KmPGK_*-*KmARO4^K221L^*-*ScCYC1*t, P*_KmPGK_*-*KmARO7^G141S^*-*ScCYC1*t, P*_KmTEF3_*-*KmPHA2*-*ScCYC1*t | This study |
| pIW1264 | pIW1234 with P*_KmPGK_*-*KmARO4^K221L^*-*ScCYC1*t, P*_KmPGK_*-*KmARO7^G141S^*-*ScCYC1*t, P*_KmPGK_*-*KmPHA2*-*ScCYC1*t | This study |
| pIW1265 | pIW1234 with P*_KmPGK_*-*KmARO4^K221L^*-*ScCYC1*t, P*_KmPGK_*-*KmARO7^G141S^*-*ScCYC1*t, P*_KmTDH3_*-*KmPHA2*-*ScCYC1*t | This study |
| pIW1266 | pIW1234 with P*_KmPGK_*-*KmARO4^K221L^*-*ScCYC1*t, P*_KmTDH3_*-*KmARO7^G141S^*-*ScCYC1*t, P*_KmTEF3_*-*KmPHA2*-*ScCYC1*t | This study |
| pIW1267 | pIW1234 with P*_KmPGK_*-*KmARO4^K221L^*-*ScCYC1*t, P*_KmTDH3_*-*KmARO7^G141S^*-*ScCYC1*t, P*_KmPGK_*-*KmPHA2*-*ScCYC1*t | This study |
| pIW1268 | pIW1234 with P*_KmPGK_*-*KmARO4^K221L^*-*ScCYC1*t, P*_KmTDH3_*-*KmARO7^G141S^*-*ScCYC1*t, P*_KmTDH3_*-*KmPHA2*-*ScCYC1*t | This study |
| pIW1269 | pIW1234 with P*_KmTDH3_*-*KmARO4^K221L^*-*ScCYC1*t, P*_KmTEF3_*-*KmARO7^G141S^*-*ScCYC1*t, P*_KmTEF3_*- *KmPHA2*-*ScCYC1*t | This study |
| pIW1270 | pIW1234 with P*_KmTDH3_*-*KmARO4^K221L^*-*ScCYC1*t, P*_KmTEF3_*- *KmARO7^G141S^*-*ScCYC1*t, P*_KmPGK_*-*KmPHA2*-*ScCYC1*t | This study |
| pIW1271 | pIW1234 with P*_KmTDH3_*-*KmARO4^K221L^*-*ScCYC1*t, P*_KmTEF3_*-*KmARO7^G141S^*-*ScCYC1*t, P*_KmTDH3_*-*KmPHA2*-*ScCYC1*t | This study |
| pIW1272 | pIW1234 with P*_KmTDH3_*-*KmARO4^K221L^*-*ScCYC1*t, P*_KmPGK_*-*KmARO7^G141S^*-*ScCYC1*t, P*_KmTEF3_*-*KmPHA2*-*ScCYC1*t | This study |
| pIW1273 | pIW1234 with P*_KmTDH3_*-*KmARO4^K221L^*-*ScCYC1*t, P*_KmPGK_*-*KmARO7^G141S^*-*ScCYC1*t, P*_KmPGK_*-*KmPHA2*-*ScCYC1t* | This study |
| pIW1274 | pIW1234 with P*_KmTDH3_*-*KmARO4^K221L^*-*ScCYC1*t, P*_KmPGK_*-*KmARO7^G141S^*- *ScCYC1*t, P*_KmTDH3_*-*KmPHA2*-*ScCYC1*t | This study |
| pIW1275 | pIW1234 with P*_KmTDH3_*-*KmARO4^K221L^*-*ScCYC1*t, P*_KmTDH3_*-*KmARO7^G141S^*- *ScCYC1*t, P*_KmTEF3_*-*KmPHA2*-*ScCYC1*t | This study |
| pIW1276 | pIW1234 with P*_KmTDH3_*-*KmARO4^K221L^*-*ScCYC1*t, P*_KmTDH3_*-*KmARO7^G141S^*-*ScCYC1*t, P*_KmPGK_*-*KmPHA2*-*ScCYC1*t | This study |
| pIW1277 | pIW1234 with P*_KmTDH3_*-*KmARO4^K221L^*-*ScCYC1*t, P*_KmTDH3_*- *KmARO7^G141S^*-*ScCYC1*t, P*_KmTDH3_*-*KmPHA2*-*ScCYC1*t | This study |
| pIW869 | pIW601 with *EAT1* targeting sgRNA | Löbs et al.^5^ |
| pIW1278 | pIW601 with *ARO8* targeting sgRNA | This study |
| pIW1279 | Replacing the P*_ScTDH3_*-*EGFP* in pIW1198 with P*_KmTEF3_*-*KmARO10* | This study |
| pIW1280 | Replacing the *EGFP* in pIW1135 with *KmADH1* | This study |
| pIW1281 | Replacing the *EGFP* in pIW1135 with *KmADH2* | This study |
| pIW1282 | Replacing the *EGFP* in pIW1135 with *KmADH5* | This study |
|  |  |  |
| Plasmids | Description | Reference (Addgene #) |
| pIW1283 | Replacing the *EGFP* in pIW1135 with *KmADH6* | This study |
| pIW1284 | Replacing the *EGFP* in pIW1135 with *KmADH7* | This study |
| pIW1285 | Replacing the *EGFP* in pIW1135 with *KmSFA1* | This study |
| pIW1286 | Replacing the *EGFP* in pIW1135 with *ScADH1* | This study |
| pIW1287 | Replacing the *EGFP* in pIW1135 with *ScADH2* | This study |
| pIW1288 | Replacing the *EGFP* in pIW1135 with *ScPAR1* | This study |
| pIW1289 | Replacing the *EGFP* in pIW1135 with *ScPAR2* | This study |
| pIW1290 | Replacing the *EGFP* in pIW1135 with *ScPAR3* | This study |
| pIW1291 | Replacing the *EGFP* in pIW1135 with *ScPAR4* | This study |
| pIW1292 | Replacing the *EGFP* in pIW1135 with *KmPAR1* | This study |
| pIW1293 | Replacing the *EGFP* in pIW1135 with *KmPAR2* | This study |
| pIW1294 | Replacing the *EGFP* in pIW1135 with *KmPAR3* | This study |
| pIW1295 | Replacing the P*_ScTDH3_* in pIW1198 with P*_KmNC1_*^7^ | This study |
| pIW1296 | Replacing the *EGFP* in pIW1295 with codon-optimized *rosePAR* | This study |
|  |  |  |
| Strains | Description | Reference |
| *E. coli* TOP 10 | F- *mcrA* Δ (*mrr*-*hsd*RMS-*mcr*BC) Φ80*lac*ZΔM15 Δ *lac*X74 *rec*A1 *ara*D139 Δ (araleu)7697 *gal*U *gal*K *rps*L (StrR) *end*A1 *nup*G | Thermo Fisher Scientific |
| YS4 | *S. cerevisiae* BY4742 | Shao et al.^6^ |
| YS626 | *K. marxianus* CBS 6556 *ura3Δ his3Δ* | Lang et al.^7^ |
| YS1155 | YS626 *ura3Δ*::P*_ScTDH3_*-*EGFP*-*ScCYC1*t | This study |
| YS1156 | YS626 *xyl2*::P*_ScTDH3_*-*EGFP*-*ScCYC1*t | This study |
| YS1157 | YS626 *abz1*::P*_ScTDH3_*-*EGFP*-*ScCYC1*t | This study |
| YS1158 | YS626 *lys1*::P*_ScTDH3_*-*EGFP*-*ScCYC1*t | This study |
| YS1159 | YS626 *sdl1*::P*_ScTDH3_*-*EGFP*-*ScCYC1*t | This study |
| YS1160 | YS626 *abz1*::P*_ScTDH3_*-*DSRED*-*ScCYC1*t | This study |
| YS1161 | YS626 *abz1*::P*_ScTDH3_*-*YFP*-*ScCYC1*t | This study |
| YS1162 | YS626 *abz1*::P*_ScTDH3_*-*DSRED*-*ScCYC1*t, P*_ScTDH3_*-*EGFP*-*ScCYC1*t | This study |
| YS1163 | YS626 *abz1*::P*_ScTDH3_*-*DSRED*-*ScCYC1*t, P*_ScTDH3_*-*EGFP*-*ScCYC1*t, P*_ScTDH3_*-*YFP*-*ScCYC1*t | This study |
| YS1164 | YS626 *abz1*::P*_ScTDH3_*-*KmARO4^K221L^*-*ScCYC1*t, P*_ScTDH3_*-*KmARO10*-*ScCYC1*t | This study |
| YS1165 | YS626 *abz1*::P*_KmTEF3_*-E*GFP*-*ScCYC1*t | This study |
| YS1166 | YS626 *abz1*::P*_KmPGK_*-*EGFP*-*ScCYC1*t | This study |
| YS1167 | YS626 *abz1*::P*_KmTDH3_*-*EGFP*-*ScCYC1*t | This study |
| YS1168 | YS626 *abz1*::P*_KmTEF3_*-*KmARO4^K221L^*-*ScCYC1*t, P*_KmTEF3_*-*KmARO7^G141S^*-*ScCYC1*t, P*_KmTEF3_*-*KmPHA2*-*ScCYC1t* | This study |
| YS1169 | YS626 *abz1*::P*_KmTEF3_*-*KmARO4^K221L^*-*ScCYC1*t, P*_KmTEF3_*- *KmARO7^G141S^*-*ScCYC1*t, P*_KmPGK_*-*KmPHA2*-*ScCYC1*t | This study |
| YS1170 | YS626 abz1::P*_KmTEF3_*-*KmARO4^K221L^*-*ScCYC1*t, P*_KmTEF3_*-*KmARO7^G141S^*-*ScCYC1*t, P*_KmTDH3_*-*KmPHA2*-*ScCYC1*t | This study |
| YS1171 | YS626 *abz1*::P*_KmTEF3_*-*KmARO4^K221L^*-*ScCYC1*t, P*_KmPGK_*-*KmARO7^G141S^*-*ScCYC1*t, P*_KmTEF3_*-*KmPHA2*-*ScCYC1*t | This study |
| YS1172 | YS626 *abz1*::P*_KmTEF3_*-*KmARO4^K221L^*-*ScCYC1*t, P*_KmPGK_*-*KmARO7^G141S^*- *ScCYC1*t, P*_KmPGK_*-*KmPHA2*-*ScCYC1*t | This study |
| YS1173 | YS626 *abz1*::P*_KmTEF3_*-*KmARO4^K221L^*-*ScCYC1*t, P*_KmPGK_*-*KmARO7^G141S^*-*ScCYC1*t, P*_KmTDH3_*-*KmPHA2*-*ScCYC1*t | This study |
| YS1174 | YS626 *abz1*::P*_KmTEF3_*-*KmARO4^K221L^*-*ScCYC1*t, P*_KmTDH3_*-*KmARO7^G141S^*-*ScCYC1*t, P*_KmTEF3_*-*KmPHA2*-*ScCYC1*t | This study |
| YS1175 | YS626 *abz1*::P*_KmTEF3_*-*KmARO4^K221L^*-*ScCYC1*t, P*_KmTDH3_*-*KmARO7^G141S^*-*ScCYC1*t, P*_KmPGK_*-*KmPHA2*-*ScCYC1*t | This study |
| YS1176 | YS626 *abz1*::P*_KmTEF3_*-*KmARO4^K221L^*-*ScCYC1*t, P*_KmTDH3_*-*KmARO7^G141S^*-*ScCYC1*t, P*_KmTDH3_*-*KmPHA2*-*ScCYC1*t | This study |
|  |  |  |
| Strains | Description | Reference |
| YS1177 | YS626 *abz1*::P*_KmPGK_*-*KmARO4^K221L^*-*ScCYC1*t, P*_KmTEF3_*-*KmARO7^G141S^*-*ScCYC1*t, P*_KmTEF3_*-*KmPHA2*-*ScCYC1*t | This study |
| YS1178 | YS626 *abz1*::P*_KmPGK_*-*KmARO4^K221L^*-*ScCYC1*t, P*_KmTEF3_*-*KmARO7^G141S^*-*ScCYC1*t, P*_KmPGK_*-*KmPHA2*- *ScCYC1*t | This study |
| YS1179 | YS626 *abz1*::P*_KmPGK_*-*KmARO4^K221L^*-*ScCYC1*t, P*_KmTEF3_*-*KmARO7^G141S^*-*ScCYC1*t, P*_KmTDH3_*-*KmPHA2*- *ScCYC1*t | This study |
| YS1180 | YS626 *abz1*::P*_KmPGK_*-*KmARO4^K221L^*-*ScCYC1*t, P*_KmPGK_*-*KmARO7^G141S^*-*ScCYC1*t, P*_KmTEF3_*-*KmPHA2*-*ScCYC1*t | This study |
| YS1181 | YS626 *abz1*::P*_KmPGK_*-*KmARO4^K221L^*-*ScCYC1*t, P*_KmPGK_*-*KmARO7^G141S^*-*ScCYC1*t, P*_KmPGK_*-*KmPHA2*-*ScCYC1*t | This study |
| YS1182 | YS626 *abz1*::P*_KmPGK_*-*KmARO4^K221L^*-*ScCYC1*t, P*_KmPGK_*-*KmARO7^G141S^*-*ScCYC1*t, P*_KmTDH3_*-*KmPHA2*-*ScCYC1*t | This study |
| YS1183 | YS626 *abz1*::P*_KmPGK_*-*KmARO4^K221L^*-*ScCYC1*t, P*_KmTDH3_*-*KmARO7^G141S^*-*ScCYC1*t, P*_KmTEF3_*-*KmPHA2*-*ScCYC1*t | This study |
| YS1184 | YS626 *abz1*::P*_KmPGK_*-*KmARO4^K221L^*-*ScCYC1*t, P*_KmTDH3_*-*KmARO7^G141S^*-*ScCYC1*t, P*_KmPGK_*-*KmPHA2*-*ScCYC1*t | This study |
| YS1185 | YS626 *abz1*::P*_KmPGK_*-*KmARO4^K221L^*-*ScCYC1*t, P*_KmTDH3_*-*KmARO7^G141S^*-*ScCYC1*t, P*_KmTDH3_*-*KmPHA2*-*ScCYC1*t | This study |
| YS1186 | YS626 *abz1*::P*_KmTDH3_*-*KmARO4^K221L^*-*ScCYC1*t, P*_KmTEF3_*-*KmARO7^G141S^*-*ScCYC1*t, P*_KmTEF3_*- *KmPHA2*-*ScCYC1*t | This study |
| YS1187 | YS626 *abz1*::P*_KmTDH3_*-*KmARO4^K221L^*-*ScCYC1*t, P*_KmTEF3_*- *KmARO7^G141S^*-*ScCYC1*t, P*_KmPGK_*-*KmPHA2*-*ScCYC1*t | This study |
| YS1188 | YS626 *abz1*::P*_KmTDH3_*-*KmARO4^K221L^*-*ScCYC1*t, P*_KmTEF3_*-*KmARO7^G141S^*-*ScCYC1*t, P*_KmTDH3_*-*KmPHA2*-*ScCYC1*t | This study |
| YS1189 | YS626 *abz1*::P*_KmTDH3_*-*KmARO4^K221L^*-*ScCYC1*t, P*_KmPGK_*-*KmARO7^G141S^*-*ScCYC1*t, P*_KmTEF3_*-*KmPHA2*-*ScCYC1*t | This study |
| YS1190 | YS626 *abz1*::P*_KmTDH3_*-*KmARO4^K221L^*-*ScCYC1*t, P*_KmPGK_*-*KmARO7^G141S^*-*ScCYC1*t, P*_KmPGK_*-*KmPHA2*-*ScCYC1*t | This study |
| YS1191 | YS626 *abz1*::P*_KmTDH3_*-*KmARO4^K221L^*-*ScCYC1*t, P*_KmPGK_*-*KmARO7^G141S^*- *ScCYC1*t, P*_KmTDH3_*-*KmPHA2*-*ScCYC1*t | This study |
| YS1192 | YS626 *abz1*::P*_KmTDH3_*-*KmARO4^K221L^*-*ScCYC1*t, P*_KmTDH3_*-*KmARO7^G141S^*- *ScCYC1*t, P*_KmTEF3_*-*KmPHA2*-*ScCYC1*t | This study |
| YS1193 | YS626 *abz1*::P*_KmTDH3_*-*KmARO4^K221L^*-*ScCYC1*t, P*_KmTDH3_*-*KmARO7^G141S^*-*ScCYC1*t, P*_KmPGK_*-*KmPHA2*-*ScCYC1*t | This study |
| YS1194 | YS626 *abz1*::P*_KmTDH3_*-*KmARO4^K221L^*-*ScCYC1*t, P*_KmTDH3_*- *KmARO7^G141S^*-*ScCYC1*t, P*_KmTDH3_*-*KmPHA2*-*ScCYC1*t | This study |
| YS1195 | YS1171 *eat1Δ* | This study |
| YS1196 | YS1171 *aro8Δ* | This study |
| YS1197 | YS1171 *ura3Δ*::P*_KmTEF3_*-*KmARO10*-*ScCYC1*t | This study |
| YS1198 | YS1197 *eat1Δ* | This study |
| YS1199 | YS1164 *ura3Δ*::P*_KmNC1_*-*rosePAR*-*ScCYC1*t | This study |

Table S3. Primers used for cloning in this study.

| Primers | Sequence (5’ to 3’, priming parts are underlined) | Use |
| --- | --- | --- |
| CasML_125 | GAATCCCGTCAGTGTCAACCTTGCCGAGTTATCGTCCAAGGGTTTTAGAGCTAGAAATAG | URA3_f_pCRISPR |
| CasML_126 | CTATTTCTAGCTCTAAAACCCTTGGACGATAACTCGGCAAGGTTGACACTGACGGGATTC | URA3_r_pCRISPR |
| CasML_045 | GAATCCCGTCAGTGTCAACCAGAATCTTGCTGTTCGAAGCGGTTTTAGAGCTAGAAATAG | ABZ1_f_pCRISPR |
| CasML_046 | CTATTTCTAGCTCTAAAACCGCTTCGAACAGCAAGATTCTGGTTGACACTGACGGGATTC | ABZ1_r_pCRISPR |
| CasML_043 | GAATCCCGTCAGTGTCAACCAAAGAGATACAGCTTCTGTTGGTTTTAGAGCTAGAAATAG | LYS1_f_pCRISPR |
| CasML_044 | CTATTTCTAGCTCTAAAACCAACAGAAGCTGTATCTCTTTGGTTGACACTGACGGGATTC | LYS1_r_pCRISPR |
| CasML_039 | GAATCCCGTCAGTGTCAACCATTTGATAATGCAACCCTCTGGTTTTAGAGCTAGAAATAG | SDL1_f_pCRISPR |
| CasML_040 | CTATTTCTAGCTCTAAAACCAGAGGGTTGCATTATCAAATGGTTGACACTGACGGGATTC | SDL1_r_pCRISPR |
| CasML_141 | GAATCCCGTCAGTGTCAACCGATCCACGACATGCAATACGGGTTTTAGAGCTAGAAATAG | ARO8_f_pCRISPR |
| CasML_142 | CTATTTCTAGCTCTAAAACCCGTATTGCATGTCGTGGATCGGTTGACACTGACGGGATTC | ARO8_f_pCRISPR |
| ML_165 | TGGAGCTCCACCGCGGTGGCCTAGATCTATTATGCATTATAATTAATAGTTGTAGC | URA3_pHDup_f |
| ML_156 | TATTGATAATGATAAACTGCGCATTAACAACCCTCTAGGTT | URA3_pHDup_r |
| ML_105 | TGGAGCTCCACCGCGGTGGCGAAGGCCAATTAGTGGAGAACTTGACAT | XYL2_pHDup_f |
| ML_092 | TATTGATAATGATAAACTGCGGGTAACTGCTTCGCCTACTTC | XYL2_pHDup_r |
| ML_039 | TGGAGCTCCACCGCGGTGGCGCCACTCGATGCGATGACTTG | ABZ1_pHDup_f |
| ML_074 | TATTGATAATGATAAACTGCGGGTGGTATTGGACCCCATAC | ABZ1_pHDup_r |
| ML_077 | TGGAGCTCCACCGCGGTGGCAACGGGAATGGTGTGTTATATGACCTTG | LYS1_pHDup_f |
| ML_070 | TATTGATAATGATAAACTGCGGGGAAGTTGTTCCAAGGAAGG | LYS1_pHDup_r |
| ML_115 | TGGAGCTCCACCGCGGTGGCTAGCAACTTTTGCTAGAAGAAAAAACTCAA | SDL1_pHDup_f |
| ML_100 | TATTGATAATGATAAACTGCGGATGAACATATAACTTTTTCGAATATGTAGATTGC | SDL1_pHDup_r |
| ML_147 | GCTCGAAGGCTTTAATTTGCGGGTCTCTAGCGCACGGTGAATA | URA3_pHDdown_f |
| ML_158 | CAAAAGCTGGGTACCGGCCGAATAAAAATTGACTCTTGTATCTTTCTTCACACTAGTAGT | URA3_pHDdown_r |
| ML_109 | GCTCGAAGGCTTTAATTTGCGTCGCTGTAGAGCCAGGTGTTC | XYL2_pHDdown_f |
| ML_094 | CAAAAGCTGGGTACCGGCCGTCAAAGCTTTGGCATTGACTTTGCCTTC | XYL2_pHDdown_r |
| ML_091 | GCTCGAAGGCTTTAATTTGCTTGGTGGGAAATTAATCCAGAATTTTATTAAATTGGCGAC | ABZ1_pHDdown_f |
| ML_078 | CAAAAGCTGGGTACCGGCCGGTACAGTTTCCCCTCCTTATGGTTAATCAAAATGC | ABZ1_pHDdown_r |
| ML_081 | GCTCGAAGGCTTTAATTTGCTGGGTCAAGGCAAGAAAGCTCTATGAGA | LYS1_pHDdown_f |
| ML_072 | CAAAAGCTGGGTACCGGCCGAGAACCCGAAGTCGAAGAAGAAGAAGAAGAAG | LYS1_pHDdown_r |
| ML_119 | GCTCGAAGGCTTTAATTTGCGGGAGGGCCATTCTGAGATGG | SDL1_pHDdown_f |
| ML_102 | CAAAAGCTGGGTACCGGCCGCACCATTCCCGAGACAAAGAGATTCTCT | SDL1_pHDdown_r |
| ML_001 | TTCTAGAACTAGTGGATCCCCCGGGATGGCTAGCATGACTGGTGGACA | EGFP_f |
| ML_002 | GACATAACTAATTACATGACTCGAGTCATTTGTATAGTTCATCCATGCCATGTGTAATCC | EGFP_r |
| ML_121 | GATTCTAGAACTAGTGGATCCCATGGACAACACCGAGGACGTC | DSRED_f |
| ML_106 | GCGTGACATAACTAATTACATGACTACTGGGAGCCGGAGTGG | DSRED_r |
| ML_013 | GATTCTAGAACTAGTGGATCCCATGGTGAGCAAAGGCGAAGA | YFP_f |
| ML_014 | GCGTGACATAACTAATTACATGACTTATAGAGCTCGTTCATGCCCTC | YFP_r |
| ML_177 | GACGGATTCTAGAACTAGTGGATCCC | DSRED-CYC1t_f |
| ML_170 | GCAAATTAAAGCCTTCGAGCGTCC | DSRED-CYC1t_r |
| ML_003 | ACGCTCGAAGGCTTTAATTTGCGGCCGCAGTTTATCATTATCAATACTCGC | P_ScTDH3__f, P_ScTDH3_-YFP_f |
| ML_004 | TCCACCAGTCATGCTAGCCATC | P_ScTDH3_-EGFP_r,  P_KmTEF3_-EGFP_r |
| ML_005 | GGCATGGATGAACTATACAAATGACTCGAGTCATGTAATTAGTTATGTCACGCT | CYC1t_f |
| ML_006 | GCAAATTAAAGCCTTCGAGCG | CYC1t_r |
| ML_016 | GAATGTAAGCGTGACATAACTAATTACATGAC | P_ScTDH3_-YFP_r |
| ML_181 | TCTAGAACTAGTGGATCCCCCGGGATGAGTGAATCTCCAATGTTCGCTGC | ScARO4_f |
| ML_174 | ACATAACTAATTACATGACTCGAGCTATTTCTTGTTAACTTCTCTTCTTTGTCTGACAGC | ScARO4_r |
| ML_183 | TCTAGAACTAGTGGATCCCCCGGGATGGATTTCACAAAACCAGAAACTG | ScARO7_f |
| ML_176 | ACATAACTAATTACATGACTCGAGTTACTCTTCCAACCTTCTTAGCAAG | ScARO7_r |
| ML_185 | TCTAGAACTAGTGGATCCCCCGGGATGTCAGCTACACCACAACCTATG | KmARO4_f |
| ML_178 | ACATAACTAATTACATGACTCGAGTTATTTAGCGGCCTTCTTTTTTAGTTCTCT | KmARO4_r |
| ML_187 | TCTAGAACTAGTGGATCCCCCGGGATGGATTTTTTTAAACCAGAAACTGTTC | KmARO7_f |
| ML_180 | ACATAACTAATTACATGACTCGAGTCATTTCTCTTCATCCTCCAAC | KmARO7_r |
| ML_239 | TTCTAGAACTAGTGGATCCCCCGGGATGGTTAAAGTGCTGTATCTAGGGCC | KmPHA2_f |
| ML_232 | GACATAACTAATTACATGACTCGAGCTAAGACACCTGGTAATACGAAGGATTTCT | KmPHA2_r |
| ML_241 | GAGGTTAAATATGTGTTCTATTTCCTCTAGACCGTTCCATGCGGACTCCCTTG | KmPHA2_S252_TCG_to_TCT_f |
| ML_234 | CAAGGGAGTCCGCATGGAACGGTCTAGAGGAAATAGAACACATATTTAACCTC | KmPHA2_S252_TCG_to_TCT_r |
| ML_219 | TTCCATGCGGACTCCCTTGC | KmPHA2_linear_f |
| ML_212 | GAACACATATTTAACCTCTGATCCTTCAATACGT | KmPHA2_linear_r |
| ML_237 | TTCTCACCATTTCATGGGTGTTACTTTGCATGGTGTTGCTGCTATCACCACTA | ScARO4^K229L^  insert_f |
| ML_230 | TAGTGGTGATAGCAGCAACACCATGCAAAGTAACACCCATGAAATGGTGAGAA | ScARO4^K229L^  insert_r |
|  |  |  |
| Primers | Sequence (5’ to 3’, priming parts are underlined) | Use |
| ML_189 | CATGGTGTTGCTGCTATCACCACTAC | ScARO4  linear_f |
| ML_182 | AGTAACACCCATGAAATGGTGAGAATGAG | ScARO4  linear_r |
| ML_225 | AGATGGTGATGATAAGAATAACTTCTCTTCTGTTGCCACTAGAGATATAGAAT | ScARO7^G141S^  insert_f |
| ML_220 | ATTCTATATCTCTAGTGGCAACAGAAGAGAAGTTATTCTTATCATCACCATCT | ScARO7^G141S^  insert_r |
| ML_223 | TCTGTTGCCACTAGAGATATAGAATGTTTG | ScARO7  linear_f |
| ML_218 | GAAGTTATTCTTATCATCACCATCTCTTTTCG | ScARO7  linear_r |
| ML_211 | CCCACATCACTTCATGGGTGTTACCTTGCACGGTGTTGCTGCCATCACCACCA | KmARO4^K221L^  insert_f |
| ML_206 | TGGTGGTGATGGCAGCAACACCGTGCAAGGTAACACCCATGAAGTGATGTGGG | KmARO4^K221L^  insert_r |
| ML_207 | CACGGTGTTGCTGCCATCAC | KmARO4  linear_f |
| ML_202 | GGTAACACCCATGAAGTGATGTGG | KmARO4  linear_r |
| ML_191 | AGAGGGAAACACATCTGAGAATTTTTCTAGTGTGGCTACGAGGGATATCGAAA | KmARO7^G141S^  insert_f |
| ML_184 | TTTCGATATCCCTCGTAGCCACACTAGAAAAATTCTCAGATGTGTTTCCCTCT | KmARO7^G141S^  insert_r |
| ML_193 | AGTGTGGCTACGAGGGATATCG | KmARO7  linear_f |
| ML_186 | AAAATTCTCAGATGTGTTTCCCTCTCTCT | KmARO7  linear_r |
| ML_265 | TCTAGAACTAGTGGATCCCCCGGGATGGCTCCAGTAGTTCTAGACGA | KmARO10_f |
| ML_254 | ACATAACTAATTACATGACTCGAGTTATTTTGGCTTACCATTTACCAACATAGC | KmARO10_r |
| ML_259 | GAAGAGAACTAAAAAAGAAGGCCGC | KmARO4^K221L^-CYC1t_f |
| ML_261 | TCGAAGGCTTTAATTTGCGCAGTTTATCATTATCAATACTCGC | P_ScTDH3_-ARO10_f |
| ML_252 | AAGCGTGACATAACTAATTACATGAC | P_ScTDH3_-ARO10_r |
| ML_245 | GGCGAATTGGAGCTCCACCG | ABZ1_upHD _f, URA3_upHD_f |
| ML_036 | GGGTGGTATTGGACCCCATACC | ABZ1_upHD _r |
| XL_001 | TATGGGGTCCAATACCACCCAACACCGATGAAGCAAAGAAG | P_KmTEF3__f |
| XL_002 | CACCAGTCATGCTAGCCATCCCGGGCTTTAATGTTACTTCTCTTGGAGTTAGAAC | P_KmTEF3__r |
| XL_003 | TATGGGGTCCAATACCACCCTTACCCTCACTCTTTCACATTAC | P_KmPGK__f |
| XL_004 | CACCAGTCATGCTAGCCATCCCGGGTTTTGTATCTTTATATAGGTAGTGTGTAT | P_KmPGK__r |
| XL_005 | TATGGGGTCCAATACCACCCCACTATATCAGGCCTCCACTATTCCA | P_KmTDH3__f |
| XL_006 | CACCAGTCATGCTAGCCATCCCGGGTGTGAATGTGTAAAAGTGTGTGTGTACTGTTG | P_KmTDH3__r |
| ML_331 | AAGAGAAGTAACATTAAAGCATGTCAGCTACACCACAACCTATG | P_KmTEF3_-KmARO4^K221L^_f |
| ML_320 | GACATAACTAATTACATGACTCGAGTTATTTAGCGG | KmARO4^K221L^_r, KmPHA2_r |
| ML_333 | AAGAGAAGTAACATTAAAGCATGGATTTTTTTAAACCAGAAACTGTTC | P_KmTEF3_-KmARO7^G141S^_f |
| ML_324 | AGCGTGACATAACTAATTACATGAC | KmARO7^G141S^_r |
| ML_303 | AAGAGAAGTAACATTAAAGCATGGTTAAAGTGCTGTATCTAGGGCC | P_KmTEF3_-KmPHA2_f |
| ML_263 | CCTATATAAAGATACAAAACATGTCAGCTACACCACAACCTATG | P_KmPGK_-KmARO4^K221L^_f |
| ML_307 | CCTATATAAAGATACAAAACATGGATTTTTTTAAACCAGAAACTGTTC | P_KmPGK_-KmARO7^G141S^_f |
| ML_297 | CCTATATAAAGATACAAAACATGGTTAAAGTGCTGTATCTAGGGCC | P_KmPGK_-KmPHA2_f |
| ML_293 | ACACTTTTACACATTCACACATGTCAGCTACACCACAACCTATG | P_KmTDH3_-KmARO4^K221L^_f |
| ML_309 | ACACTTTTACACATTCACACATGGATTTTTTTAAACCAGAAACTGTTC | P_KmTDH3_-KmARO7^G141S^_f |
| ML_311 | ACACTTTTACACATTCACACATGGTTAAAGTGCTGTATCTAGGGCC | P_KmTDH3_-KmPHA2_f |
| ML_007 | GGCATGGATGAACTATACAAATGAC | CYC1t_611bp_ABZ1_downHD_f |
| ML_192 | AGGGAACAAAAGCTGGTACCGGCCGGACCGTAAACTGACCCATTTCATATC | 611bp_ABZ1_downHD_r |
|  |  |  |
| Primers | Sequence (5’ to 3’, priming parts are underlined) | Use |
| ML_215 | CTGGCCCCAGTGCTGCAATGATACCGCGACTCCCACGCTCACCGGCTCCAGATTTATCA | AmpR_S240_TCT_to_AGT_insert_f |
| ML_210 | TGATAAATCTGGAGCCGGTGAGCGTGGGAGTCGCGGTATCATTGCAGCACTGGGGCCAG | AmpR_S240_TCT_to_AGT_insert _r |
| ML_213 | TATCATTGCAGCACTGGGGC | 611bp_ABZ1_downHD_linear_f |
| ML_208 | TCACCGGCTCCAGATTTATCAGC | 611bp_ABZ1_downHD_linear_r |
| ML_233 | GGGTCCAATACCACCCTACCGCGAGACCCACGGGTCTCTGAATCTTGGTGGGAAATTAAT | BsaI(2)_insert_f |
| ML_226 | ATTAATTTCCCACCAAGATTCAGAGACCCGTGGGTCTCGCGGTAGGGTGGTATTGGACCC | BsaI(2)_insert_r |
| ML_231 | TTGGTGGGAAATTAATCCAGAATTTTATTAAATTGGCG | 611bp_ABZ1_downHD_AmpR_S240_TCT_to_AGT_linear_f |
| ML_298 | GGGTGGTATTGGACCCCATAC | 611bp_ABZ1_downHD_AmpR_S240_TCT_to_AGT_linear_r |
| ML_377 | GGGTCCAATACCACCCTAACACCGATGAAGCAAAGAAGTAACAGC | P_KmTEF3_-KmARO4^K221L^-CYC1t_f |
| ML_366 | CGAATGTCTGTTCGACACTGGCAAATTAAAGCCTTCGAGCGTCC | KmARO4^K221L^-CYC1t_r |
| ML_365 | CAGTGTCGAACAGACATTCGAACACCGATGAAGCAAAGAAGTAACAGC | P_KmTEF3_-KmARO7^G141S^-CYC1t_f |
| ML_368 | TCGATACTGGTACTAATGCGGCAAATTAAAGCCTTCGAGCGTCC | KmARO7^G141S^-CYC1t_r |
| ML_371 | CGCATTAGTACCAGTATCGAAACACCGATGAAGCAAAGAAGTAACAGC | P_KmTEF3_-KmPHA2-CYC1t_f |
| ML_370 | GTCGCCAATTTAATAAAATTCTGGATTAATTTCCCACCAA | KmPHA2-CYC1t_r |
| ML_373 | CGCATTAGTACCAGTATCGATTACCCTCACTCTTTCACATTACCCTCC | P_KmPGK_-KmPHA2-CYC1t_f |
| ML_375 | CGCATTAGTACCAGTATCGACACTATATCAGGCCTCCACTATTCCAGG | P_KmTDH3_-KmPHA2-CYC1t_f |
| ML_367 | CAGTGTCGAACAGACATTCGTTACCCTCACTCTTTCACATTACCCTCC | P_KmPGK_-KmARO7^G141S^-CYC1t_f |
| ML_369 | CAGTGTCGAACAGACATTCGCACTATATCAGGCCTCCACTATTCCAGG | P_KmTDH3_-KmARO7^G141S^-CYC1t_f |
| ML_379 | GGGTCCAATACCACCCTTTACCCTCACTCTTTCACATTACCCTCC | P_KmPGK_-KmARO4^K221L^-CYC1t_f |
| ML_381 | GGGTCCAATACCACCCTCACTATATCAGGCCTCCACTATTCCAGG | P_KmTDH3_-KmARO4^K221L^-CYC1t_f |
| ML_238 | GCATTAACAACCCTCTAGGTTCTTTCGTAACTTC | URA3_upHD_r |
| ML_247 | ACCTAGAGGGTTGTTAATGCAACACCGATGAAGCAAAGAAGTAACAGC | URA3_upHD-P_KmTEF3__f |
| ML_015 | TCTAGAACTAGTGGATCCCCCGGGATGGCTATTCCAGAAACTCAA | KmADH1_f |
| ML_018 | ACATAACTAATTACATGACTCGAGTTATTTGGAAGTGTCAACGACAA | KmADH1_r |
| ML_017 | TCTAGAACTAGTGGATCCCCCGGGATGTCTATTCCAACTACTCAAAAGG | KmADH2_f |
| ML_020 | ACATAACTAATTACATGACTCGAGTTATTTGGAAGTGTCAACAACGT | KmADH2_r |
| ML_019 | TCTAGAACTAGTGGATCCCCCGGGATGTTTCATAGAAGAGCATTGAAG | KmADH5_f |
| ML_022 | ACATAACTAATTACATGACTCGAGTTAGCATTCATAGGCCTGTCTGA | KmADH5_r |
| ML_021 | TCTAGAACTAGTGGATCCCCCGGGATGTCCTACCCAGATAGTTTCC | KmADH6_f |
| ML_024 | ACATAACTAATTACATGACTCGAGTTATTTTTGAGCCTTGAACTCTCC | KmADH6_r |
| ML_023 | TCTAGAACTAGTGGATCCCCCGGGATGTTTCGTAAGGTCACATCTG | KmADH7_f |
| ML_026 | ACATAACTAATTACATGACTCGAGTTAAAAGTTAATAATAAGTTTCATAGCCTTTT | KmADH7_r |
| ML_025 | TCTAGAACTAGTGGATCCCCCGGGATGTCATCCGAAACCGCAGG | KmSFA1_f |
| ML_028 | ACATAACTAATTACATGACTCGAGTTATTTCTCCAAATCCAAGACGG | KmSFA1_r |
| ML_027 | TCTAGAACTAGTGGATCCCCCGGGATGTCTATCCCAGAAACTCAAAA | ScADH1_f |
| ML_030 | ACATAACTAATTACATGACTCGAGTTATTTAGAAGTGTCAACAACGTATCTAC | ScADH1_r |
| ML_029 | TCTAGAACTAGTGGATCCCCCGGGATGTCTATTCCAGAAACTCAAAAAG | ScADH2_f |
| ML_032 | ACATAACTAATTACATGACTCGAGTTATTTAGAAGTGTCAACAACGTATCTACC | ScADH2_r |
| ML_269 | TCTAGAACTAGTGGATCCCCCGGGATGACTACTGAAAAAACCGTTGTTTTTGTTTCTGG | ScPAR1_f |
|  |  |  |
| Primers | Sequence (5’ to 3’, priming parts are underlined) | Use |
| ML_258 | ACATAACTAATTACATGACTCGAGTTAGCTTTTACTTTGAACTTCTAGTAATTGCGAGGC | ScPAR1_r |
| ML_271 | TCTAGAACTAGTGGATCCCCCGGGATGTCTAATACAGTTCTAGTTTCTGGCGCTTC | ScPAR2_f |
| ML_260 | ACATAACTAATTACATGACTCGAGTTATAATCTGTTCTGCTTCTTCAAAATTTGGGCAG | ScPAR2_r |
| ML_273 | TCTAGAACTAGTGGATCCCCCGGGATGACTACTGATACCACTGTTTTCGTTTCTGG | ScPAR3_f |
| ML_262 | ACATAACTAATTACATGACTCGAGTTAGGCTTCATTTTGAACTTCTAACATTTGCGC | ScPAR3_r |
| ML_275 | TCTAGAACTAGTGGATCCCCCGGGATGTCAGTTTTCGTTTCAGGTGCTAACG | ScPAR4_f |
| ML_264 | ACATAACTAATTACATGACTCGAGTTATATTCTGCCCTCAAATTTTAAAATTTGGGAGGC | ScPAR4_r |
| ML_277 | TCTAGAACTAGTGGATCCCCCGGGATGACGTACGTTGTGGTTACTGGTG | KmPAR1_f |
| ML_266 | ACATAACTAATTACATGACTCGAGTTAGTTGTTAGCCTTTAGTATTTGGGCAACAGTATC | KmPAR1_r |
| ML_279 | TCTAGAACTAGTGGATCCCCCGGGATGACATATACAGTGGTGACAGGCG | KmPAR2_f |
| ML_268 | ACATAACTAATTACATGACTCGAG TACTTACCCACGGTACGCGC | KmPAR2_r |
| ML_281 | TCTAGAACTAGTGGATCCCCCGGGATGTCATACACGGTAATTACAGGCGC | KmPAR3_f |
| ML_270 | ACATAACTAATTACATGACTCGAGTTAATTCTGAGGAATTCCCCTGACTCTCAAAAG | KmPAR3_r |
| ML_425 | ACCTAGAGGGTTGTTAATGCCCACGCAGTGTGAATGGACTTT | URA3_upHD-P_KmNC1__f |
| ML_414 | CACCAGTCATGCTAGCCATCCCGGGTTTTGATTTGTGTTTAAGCGAGTGACTGAAG | URA3_upHD-P_KmNC1__f |

Table S4. Sequences of *KmARO4*, *KmARO4^K221L^*, *KmARO7*, *KmARO7^G141S^*, *KmPHA2*, *KmARO10*, and the codon-optimized *rosePAR*

| ***KmARO4*** |
| --- |
| ATGTCAGCTACACCACAACCTATGTTCCATGAACAAGAAGACGTTAGAATCTTGGGTTACGATCCATTAGTGTCACCAGCACTACTTCAGGCCCAAGTTCCAGCTTCTCCTGAATGTCTAGCAACAGCTCAAAGAGGTAGAAAGGAGTCTGTTGATATTATTACCGGTAAAGATGACAGAGTGCTGGTTATCGTCGGTCCATGCTCCATCCACGATTTGGATCAAGCACAAGAGTACGCTAAGATGTTGAAGGCTCTTTCGGATGAGTTGAAGGACGATTTGTGTATCATAATGAGAGCTTACTTAGAAAAGCCAAGAACCACTGTTGGCTGGAAGGGTTTGATCAACGATCCAGATGTCGACAACACTTTCAACATCAACAAAGGTTTGCAAGTATCCAGACAATTGTTTGTAAATTTGACCAGTCTGGGATTACCAATTGGTTCTGAGATGTTGGACACTATCTCTCCTCAATTCTTGGCTGATTTGCTATCTTTCGGTGCCATTGGTGCTAGAACTACCGAGTCTCAATTGCACAGAGAATTGGCCTCTGGTTTATCCTTCCCAGTCGGTTTCAAGAACGGTACCGACGGTACATTGGGTGTTGCCGTCGATGCTGTCCAAGCTGCCTCTCACCCACATCACTTCATGGGTGTTACCAAGCACGGTGTTGCTGCCATCACCACCACCAAGGGTAATGAACACTGCTTCGTTATTCTAAGAGGTGGTAAGAAGGGAACAAACTACGACCCTGCTTCCGTCGCTGAAGCCAAGGCTCAGTTGCCTGAAAAGGGTGTTCTAATGATCGACTACTCCCACGGTAACTCCAACAAGGACTTCAGAAACCAACCTAAGGTTAACGATGTCGTCTGTGAACAAATTGCCAATGGTGAAGATAAGATCATCGGTGTCATGATCGAATCTAACATCAACGAAGGTAAGCAATGCATTCCTCCAGAAGGTAAGGCTGGCTTGAAATACGGTGTCTCCATCACTGACGGATGCATAAGTTTCGAAACAACCACCGAGGTCCTACGTAAGCTAGCTGCTGCCGTAAGAGCTAGAAGAGAACTAAAAAAGAAGGCCGCTAAATAA |
| ***KmARO4^K221L^*** |
| ATGTCAGCTACACCACAACCTATGTTCCATGAACAAGAAGACGTTAGAATCTTGGGTTACGATCCATTAGTGTCACCAGCACTACTTCAGGCCCAAGTTCCAGCTTCTCCTGAATGTCTAGCAACAGCTCAAAGAGGTAGAAAGGAGTCTGTTGATATTATTACCGGTAAAGATGACAGAGTGCTGGTTATCGTCGGTCCATGCTCCATCCACGATTTGGATCAAGCACAAGAGTACGCTAAGATGTTGAAGGCTCTTTCGGATGAGTTGAAGGACGATTTGTGTATCATAATGAGAGCTTACTTAGAAAAGCCAAGAACCACTGTTGGCTGGAAGGGTTTGATCAACGATCCAGATGTCGACAACACTTTCAACATCAACAAAGGTTTGCAAGTATCCAGACAATTGTTTGTAAATTTGACCAGTCTGGGATTACCAATTGGTTCTGAGATGTTGGACACTATCTCTCCTCAATTCTTGGCTGATTTGCTATCTTTCGGTGCCATTGGTGCTAGAACTACCGAGTCTCAATTGCACAGAGAATTGGCCTCTGGTTTATCCTTCCCAGTCGGTTTCAAGAACGGTACCGACGGTACATTGGGTGTTGCCGTCGATGCTGTCCAAGCTGCCTCTCACCCACATCACTTCATGGGTGTTACCTTGCACGGTGTTGCTGCCATCACCACCACCAAGGGTAATGAACACTGCTTCGTTATTCTAAGAGGTGGTAAGAAGGGAACAAACTACGACCCTGCTTCCGTCGCTGAAGCCAAGGCTCAGTTGCCTGAAAAGGGTGTTCTAATGATCGACTACTCCCACGGTAACTCCAACAAGGACTTCAGAAACCAACCTAAGGTTAACGATGTCGTCTGTGAACAAATTGCCAATGGTGAAGATAAGATCATCGGTGTCATGATCGAATCTAACATCAACGAAGGTAAGCAATGCATTCCTCCAGAAGGTAAGGCTGGCTTGAAATACGGTGTCTCCATCACTGACGGATGCATAAGTTTCGAAACAACCACCGAGGTCCTACGTAAGCTAGCTGCTGCCGTAAGAGCTAGAAGAGAACTAAAAAAGAAGGCCGCTAAATAA |
| ***KmARO7*** |
| ATGGATTTTTTTAAACCAGAAACTGTTCTAAATTTGCAGAACATTAGAGATGAGCTCGTGAAGATGGAAGATACGATCATCTTCAATTTCATTGAGAGATCACACTTTGCCACATGTCCCAGTGTGTACCAAAACAAAGATCCATTAGTGAATCTCCCTGACTTTGATGGTAGTTTCTTAGATTGGGCCTTAATGCATGTGGAAATTGTGCAATCTCAATTGAGACGGTTCGAGTCACCAGACGAAACACCCTTCTTTCCAGATAAAATTCTCAAGCCAATCATTCCTAGCTTGAACTACCCCAAGATTCTGGCGTCCTATTCGAGTCAAATAAACTATACTGACAAAATCAAGAAGATTTACATCGAAACTATTGTACCCTTGATATCCAAGAGAGAGGGAAACACATCTGAGAATTTTGGGAGTGTGGCTACGAGGGATATCGAAACCTTGCATGCTCTAAGCAGAAGGATTCACTTTGGTAAATTTGTAGCTGAAGCAAAGTTTCAAAGTGAAAAGGAAAAATACACTGAGCTTATCCGTAACAAAGATACAGAAGGAATTATGAAGGCTATCACTAACTCTGCAGTAGAGGAGAAAATTTTGAAAAGACTACAAGTCAAAGCCGAAGTCTACGGTGTTGACCCTACTAATGCACAGGGCGATAGAAAGATCACACCTGAGTATTTGGTTAGGATTTATAAAGAAATAGTTATCCCCATTACAAAAGAGGTAGAAGTCGAATATCTTTTGAGGAGGTTGGAGGATGAAGAGAAATGA |
| ***KmARO7^G141S^*** |
| ATGGATTTTTTTAAACCAGAAACTGTTCTAAATTTGCAGAACATTAGAGATGAGCTCGTGAAGATGGAAGATACGATCATCTTCAATTTCATTGAGAGATCACACTTTGCCACATGTCCCAGTGTGTACCAAAACAAAGATCCATTAGTGAATCTCCCTGACTTTGATGGTAGTTTCTTAGATTGGGCCTTAATGCATGTGGAAATTGTGCAATCTCAATTGAGACGGTTCGAGTCACCAGACGAAACACCCTTCTTTCCAGATAAAATTCTCAAGCCAATCATTCCTAGCTTGAACTACCCCAAGATTCTGGCGTCCTATTCGAGTCAAATAAACTATACTGACAAAATCAAGAAGATTTACATCGAAACTATTGTACCCTTGATATCCAAGAGAGAGGGAAACACATCTGAGAATTTTTCTAGTGTGGCTACGAGGGATATCGAAACCTTGCATGCTCTAAGCAGAAGGATTCACTTTGGTAAATTTGTAGCTGAAGCAAAGTTTCAAAGTGAAAAGGAAAAATACACTGAGCTTATCCGTAACAAAGATACAGAAGGAATTATGAAGGCTATCACTAACTCTGCAGTAGAGGAGAAAATTTTGAAAAGACTACAAGTCAAAGCCGAAGTCTACGGTGTTGACCCTACTAATGCACAGGGCGATAGAAAGATCACACCTGAGTATTTGGTTAGGATTTATAAAGAAATAGTTATCCCCATTACAAAAGAGGTAGAAGTCGAATATCTTTTGAGGAGGTTGGAGGATGAAGAGAAATGA |
| ***KmPHA2*** |
| ATGGTTAAAGTGCTGTATCTAGGGCCTGCTGGCACATACTCACACCAAGCTGTGCTGCAACAATTCGCCGATGAGGAACTAATTCCAACGAATTCAATCCCTTCTTGCTTCAAAACGCTCATTGAAGATGAAGATATAGACTATGGGGTTGTGCCATTGGAGAATTCAACGAATGGACAAGTTGTGTTCACATACGATCTTTTCAGAGATTTTATGCAGTCCGAGGAGGAACCAAAGCTTGAAGTTGTTGGGGAACAATACGTTGATATCGCACATTGTCTCATCGCACCAGCACCATTGGAGGTAGACAAGCTCAGAAAAATCGGCACAATATATTCGCATCCACAGGTGTGGGGTCAAGTGAAGGACTATCTTGCTGATCTTGAGAAGAAGTACGGGAAGTTTACAAAAATTGACTGTAATTCCACATCTGAGGCTGTAACGAAATGCATTCAAGAGTGGGATTCAAAAGAAAGGATAACTCTTGCCATTGGAAGTCGTGCTAGTGCAAAATTAAACAAAGGTTTCATAGTAGATAGTGGAATTAATGACATAAAGGGGAATACAACAAGGTTTTTGATATTGAAAAGGCGTAAGCCGAATAATCGACTAGTGACAGATTGTCTACCTCCCAACACGGACAGTAAGAAGGTGAATTTAGTGACCTTTGTAACCAAACAGGACGACCCAGGTTCTTTGGTGGACGTTTTAAACGTATTGAAGGATCAGAGGTTAAATATGTGTTCTATTTCCTCTAGACCGTTCCATGCGGACTCCCTTGCAGGTCGAAAATGGCAGTACTGTTTCTTTATTGAGTTTTATCATAGTGAAACAACGGACTATGAGTTGTTAATGGAGAAGTTTGACGAGTACTGTGCGAAATGGATTCATTGGGGCAGATTCTACAGAAATCCTTCGTATTACCAGGTGTCTTAG |
| ***KmARO10*** |
| ATGGCTCCAGTAGTTCTAGACGATAAATCTGCATCTGAATCACCAAGAAGCGACTCTCCAGTCCACGGCCTCTCCAGTGTTGTCAAGGACATCACTCTTGGTCGTTATGTGTTCGAGAGATTGCTCAACTGTGGTTCCAAGACCATTTTCGGTGTTCCAGGTGACTTCAACTTGCCTTTGCTAGAATACTTGTACGAAGAAGAGTTGGTGCAAAACGGGCTCCAATGGGTTGGTACTTGTAACGAATTGAACGCTGCATATGCTGCGGACGGTTACTCCCGTTACACTAGTAAAATCGGTTGTGTAATCACCACATTTGGTGTCGGTGAATTGTCCGCTTTGAACGGTATTTCTGGTGCCTTTGCGGAAGATGTTAAGGTCTTGCACATTGTCGGTGTCAGTCCAACCAAGTTCAGAAAGAATGACAAGTTCCGTTCCCACAATGTTCACCACTTGGTTCCAGACTTGGATGGTGACAAGGAACCAAACCACGAAGTTTACTTCGATATGATTAAGGACAGAGTTTCTTGCTCTAGCGCTTTCTTGCACGATGTCAAATCTGCACCAGAAAAGATCGACAAGGTTATTGCTGATATTTACAAGTACAGTAAGCCAGGTTACATCTTTATTCCAGCTGATTTCGCTGATGAAATGGTCTCTAATAAGAACTTGGTTGAGACCCCAGTTATAGACTTGCCTTATGTCATTGAAAACACAACCTCTAAGGATGCTACCAAAAAGGCCGGTGACAAAATCTTGCAATGGCTATACGAATCTAAGACTCCATCTGTCTTTTCCGACGCTCTAGTTGGTAGATTTAACTTGAACAAGGATATTCGTGAGCTAATCAACAAGGTTGACATGTGGAACTTCACCACTGCCATGGCTAAGTCTTCTTTGGACGAACATCACCCAAAGCATTTGGGTGTCTACAAGGGTGCTGAAACTGGTAAGGAAATGCAATCCATTGTGGAAATGAGTGACTTGATCCTACACTTTGGTCCTTGTAAGAATGAAATCAACTTTGGTTACTACACTTTCCGTTACAACGACAACGCCAGAGTTGTTGAACTTTCGAAGGACAAGATCACATTCTTCGAAACTAAGGCTAATGGTTCAGTTGAAGTTCAAGAAGCTAACTTTGCTGCCGTTTTGAAATACATGAATGAAAACCTAGATGTCTCCAAGATTTCCACCGCTTACCCATCTGTCTCTAGAACTAGAGAACACATCGAATTTGGTGAAGATGATGAAATTAGTCAACAGAGTTTGAAGCGTATAGTGGAGAATTTCTACAACCCTGGTGATGTTTTGGTTGTTGAAACTGGTTCTTTCCAATTTAACTTGATCAACATGAAATTCGCTCCAGAAATGAAATATATGACACAAACATTCTACCTTTCTATTGGTATGGCTTTGCCAGCTGCATTAGGTGTCGGCTGTGGTATGCGCGACTACCCAAGATCTCACATTATCAACCAATCTGCTGTCCCAGCAGACTATGTTCCAAAGTTGATCTTGTGTGAAGGTGATGGTGCTGCTCAAATGACTATTCAAGAATTTGCTTCTTACATTCGTTACAAGATCCCAATGAACATTCTTTTGTTCAACAACAATGGTTACACTATTGAAAGAGCTATTCTAGGACCAACCAGAAGTTATAATGATATTGCACCAGTTAAGTGGACCGCTTTGCTTAACGCTTTTGGTGACTTTGAAAACAAATTCAGTGAAACTGTTACTGTTTCCAAGAATAAGGAAATCATTGAAGTTTTGAACGAATGGAAAAAGGAGAAGGTTCCTTCTAAGATCAAGTTGGCAGAAGTTATGTTACCAGTTATGGATATTCCTTCTGAGTTGGATGCTATGTTGGTAAATGGTAAGCCAAAATAA |
| **codon-optimized *rosePAR*** |
| ATGTCTAACAAGGTTGTTTGTGTTACCGGTGCATCTGGTTACATAGCAAGTTGGCTTGTTAAGCTTCTTCTTCAACGAGGTTACACCGTTAAGGCATCTGTGCGAAACCCAAACGATCCAACCAAGACCGAACACTTGCTTGCACTTGATGGTGCAAAGGAGAGACTTCAACTTTTCAAGGCAGATCTTTTGGAAGAAGGTTCTTTCGATTCTGCAGTTGAAGGTTGTGAAGGTGTTTTCCACACCGCATCTCCATTCTACCACGATGTTACCGATCCAAAGGCAGAACTTCTTGATCCAGCAGTTAAGGGTACCCTTAACGTTCTTAACTCTTGTTCTAAGTCTCCATCTATAAAGCGAGTTGTTTTGACCTCTTCTATAGCAGCAGTTGCATACAACGGTAAGCCACGAACCCCAGATGTCGTGGTTGATGAAACCTGGTTCACCGATCCAGATGTTTGTAAGGAATCTAAGCTTTGGTACGTTCTTTCTAAGACCTTGGCAGAAGATGCAGCATGGAAGTTCGTTAAGGAAAAGGGTATAGATATGGTTACCATAAACCCAGCAATGGTTATAGGTCCACTTTTGCAACCAACCCTTAACACCTCTGCTGCAGCAATACTTAACATAATAAAGGGTGCACGAACCTACCCAAACGCATCTTTCGGTTGGATAAACGTTAAGGATGTTGCAAACGCACACGTTCAAGCATTCGAAATACCATCTGCATCTGGTAGATACTGTCTTGTTGAACGAGTTGCACACTTCACCGAAGTTCTTCAAATAATACACGAACTTTACCCAGATTTGCAACTTCCAGAAAAGTGTTCTGATGATAAGCCATTCGTTCCAACCTACCAAGTTTCTAAGGAAAAGGCAAAGTCTTTGGGTATAGAATTCATACCACTTGATATATCTCTTAAGGAAACCATAGAATCTTTGAAGGAAAAGTCTATAGTTTCTTTCTGA |


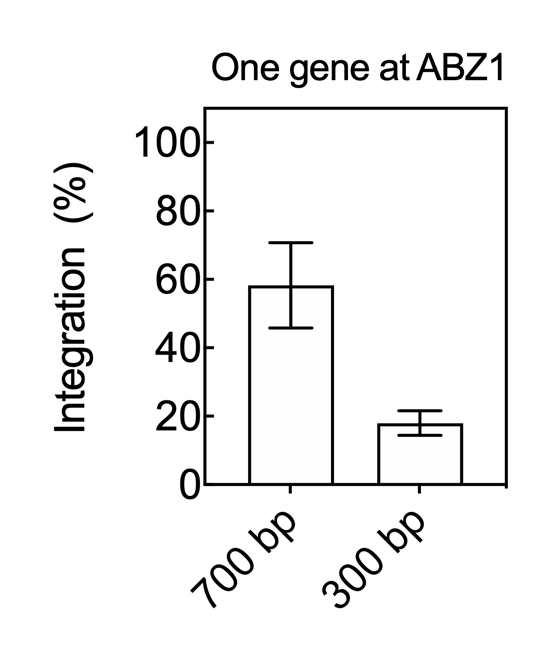


Figure S1. Homology donor length effects on the CRISPR-mediated gene integration in *K. marxianus* CBS 6556 *ura3Δ his3Δ*. All experiments were performed in biological triplicates. Bars represent the arithmetic mean, and error bars represent the standard deviation.


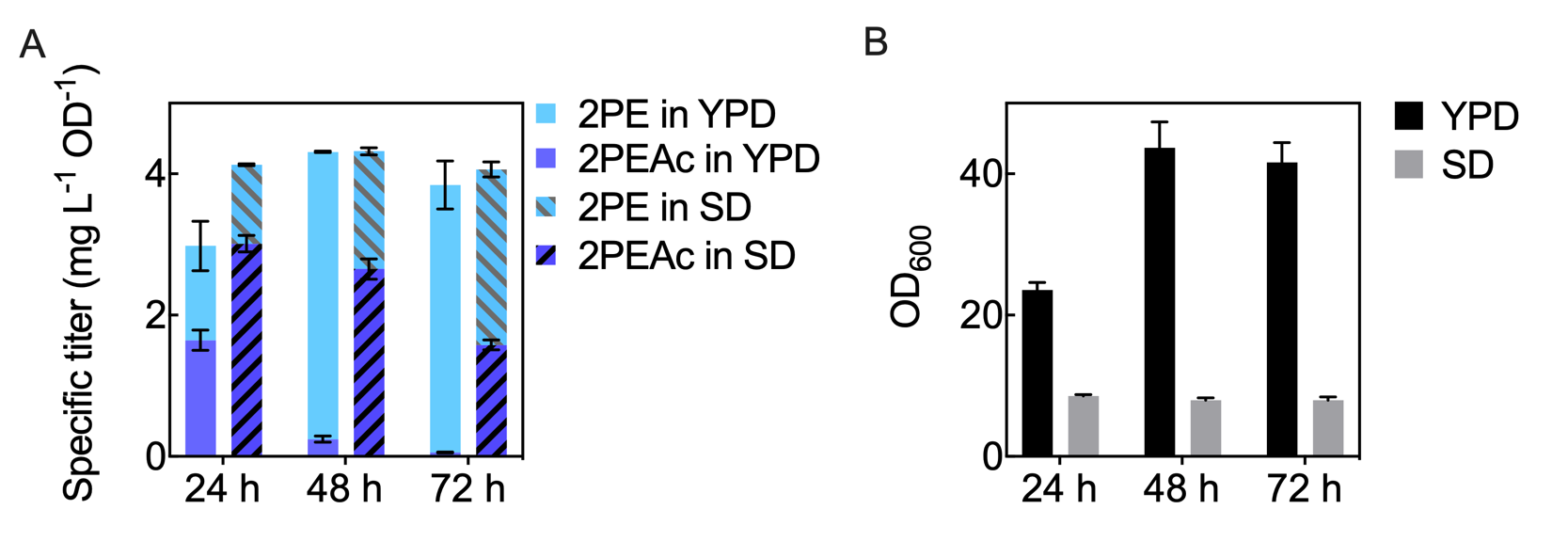


Figure S2. Medium effects on 2-PE acetylation and biomass accumulation of *K. marxianus* CBS 6556 *ura3Δ his3Δ*. (A) Specific titer of 2-PE and 2-PEAc after 24, 48, and 72 h. (B) OD_600_ at each time point for extracellular 2-PE and 2-PEAc measurement. The strain was cultivated in 25 mL YPD or synthetic defined medium with complete supplement mixture (SD) with 20 g/L D-glucose for biological triplicates at 30 °C from an initial OD_600_ of 0.05. All experiments were performed in biological triplicates. Bars and dots represent the arithmetic mean, while error bars represent the standard deviation.


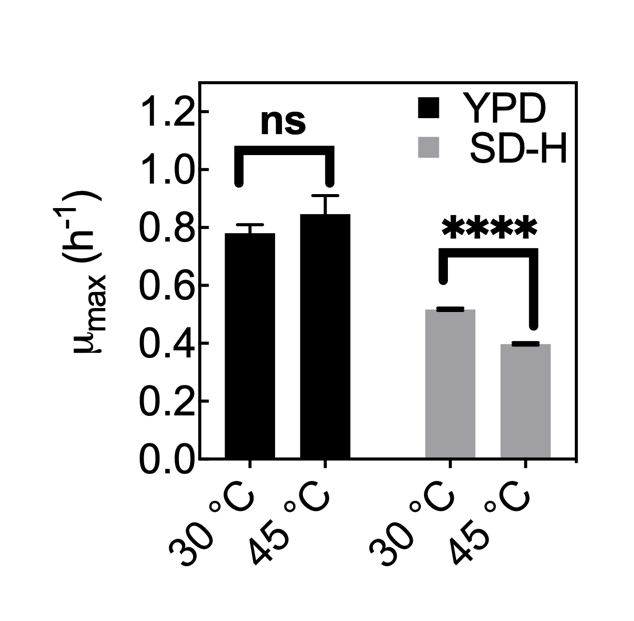


Figure S3. Undesirable effects of plasmid reliance on cell growth at elevated temperature for *K. marxianus* CBS 6556 *ura3Δ his3Δ*. The strain was inoculated in 25 mL YPD or SD-H with 20 g/L D-glucose at an initial OD_600_ of 0.05 and was cultivated at 30 °C shaker till the end of exponential phase. Statistically significant difference indicated with “ns” for P value > 0.12, and “****” for P value < 0.0001 in t tests. All experiments were performed in biological triplicates. Bars represent the arithmetic mean, and error bars represent the standard deviation.


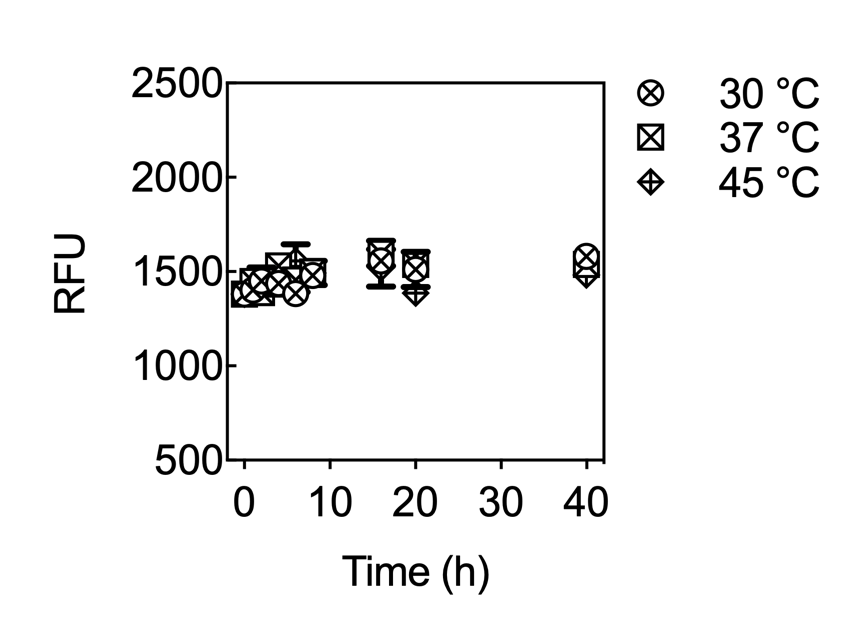


Figure S4. EGFP stability over 40 h incubation at 30, 37, and 45 °C. The *K. marxianus* strain harboring *EGFP* expression plasmid (P*_KmTEF3_*-*EGFP*-*ScCYC1*t) was cultured in 25 mL SD-H medium at 30 °C from an initial OD_600_ of 0.05. After 14 h, cells were harvested by centrifugation at 5000 g for 10 min at 4 °C. Supernatants were discarded and cell pellets were washed and resuspended with 100 ml PBS. The cell resuspension was lysed by sonication for 90 s. The sonication process was carried out on ice, and there was a 7 s pause for every 3 s sonication. After centrifugation at 11,000 g for 30 min at 4 °C, the lysate supernatant was collected and incubated. The 20× diluent was then measured at 0, 1, 2, 4, 6, 8, 16, 20, 40 hours for relative green fluorescence intensity by BioTek^®^Synergy^™^ Neo2 multi-mode microplate Reader (Ex/Em 488/511). PBS was used as the blank of measurement, and all data was background subtracted.


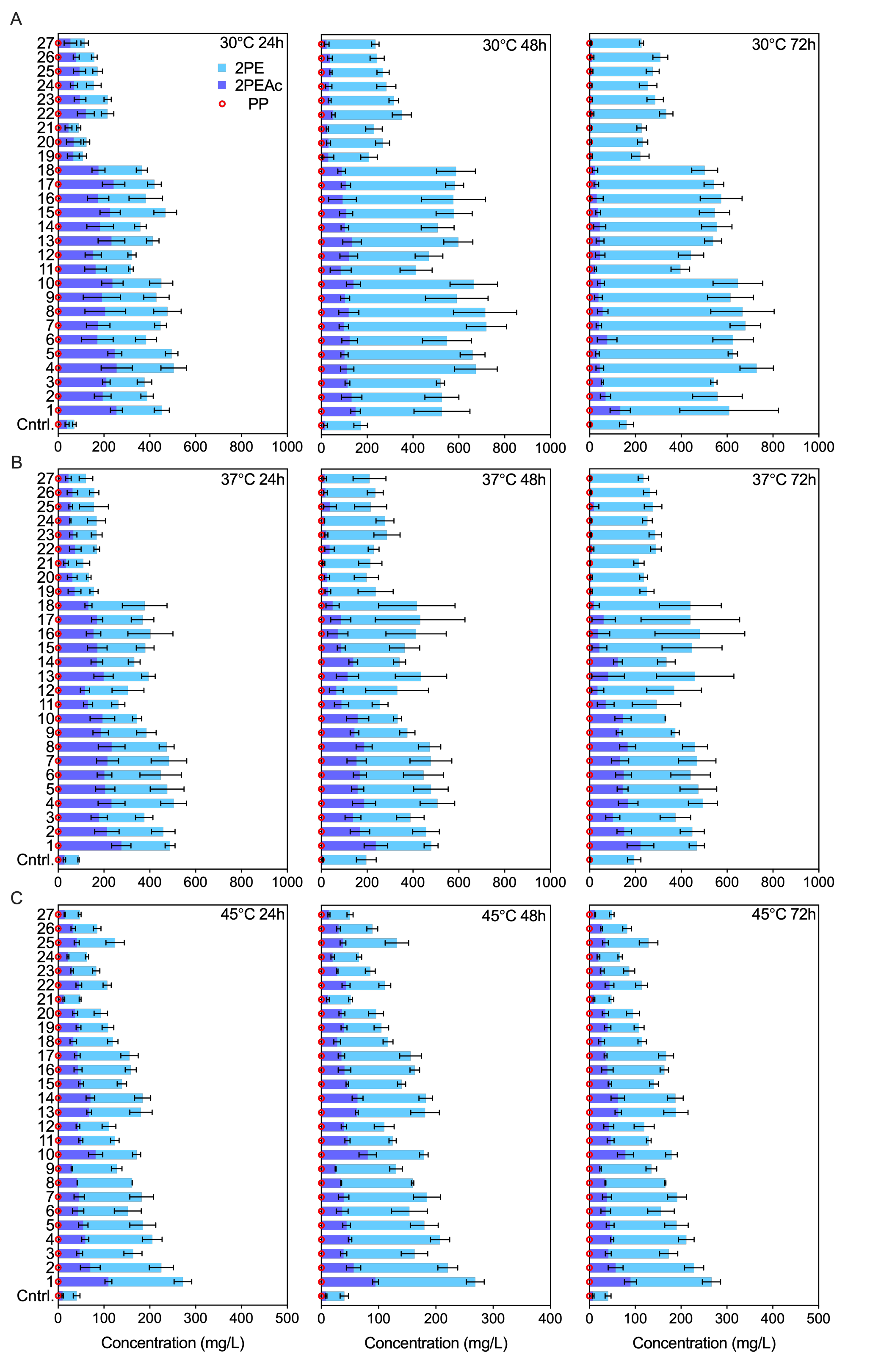


Figure S5. Extracellular formation of 2-phenylethanol (2-PE), 2-phenylethyl acetate (2-PEAc), and phenylpyruvate (PP) after 24, 48, and 72 h cultivation at (A) 30, (B) 37, and (C) 45 °C. Cell cultures were initiated from an OD_600_ of 0.05 in 25 mL YPD medium with 20 g/L glucose. The strain Cntrl. was *K. marxianus* CBS 6556 *ura3Δ his3Δ* as the negative control. Numbers 1 to 27 denoted corresponding gene overexpression combinations integrated into the genome of Cntrl. All experiments are performed at least in biological triplicates. Bars and dots represent arithmetic means, and error bars represent standard deviations.


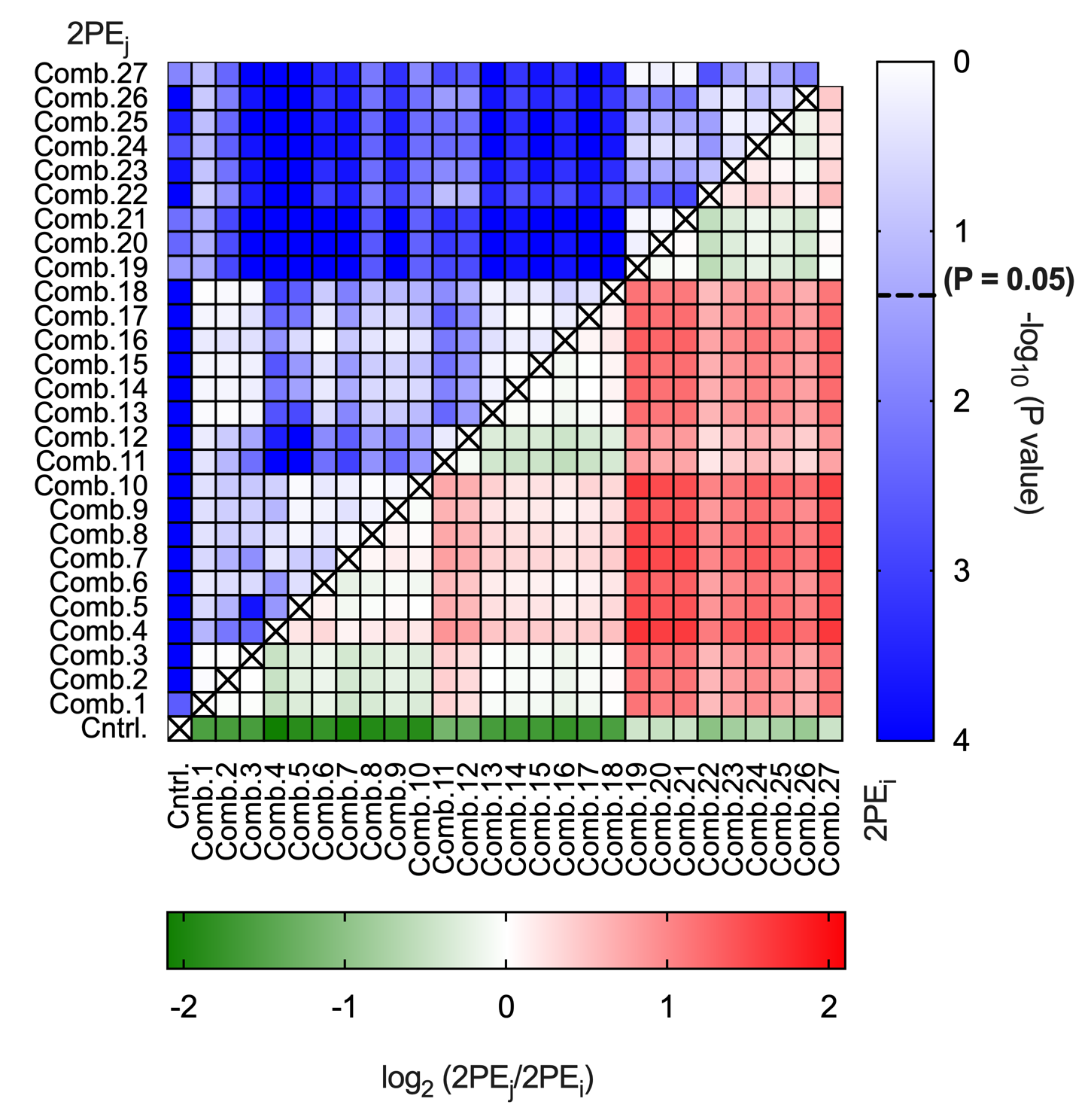


Figure S6. Effects of varied expression combinations of *KmARO4^K221L^*, *KmARO7^G141S^*, and *KmPHA2* on 2-PE accumulation after 72 h cultivation at 30 °C. Cell cultures were initiated from an OD_600_ of 0.05 in 25 mL YPD medium with 20 g/L glucose. The strain Cntrl. was *K. marxianus* CBS 6556 *ura3Δ his3Δ* as the negative control. Comb. 1 to 27 denoted the 3^3^-factorial gene overexpression combination library integrated into the genome of Cntrl. The fold change (denoted by log_2_(2PE_j_/2PE_i_)) and the significance of difference (denoted by -log_10_(P value)) were calculated by the arithmetic means of at least triplicate biological samples.


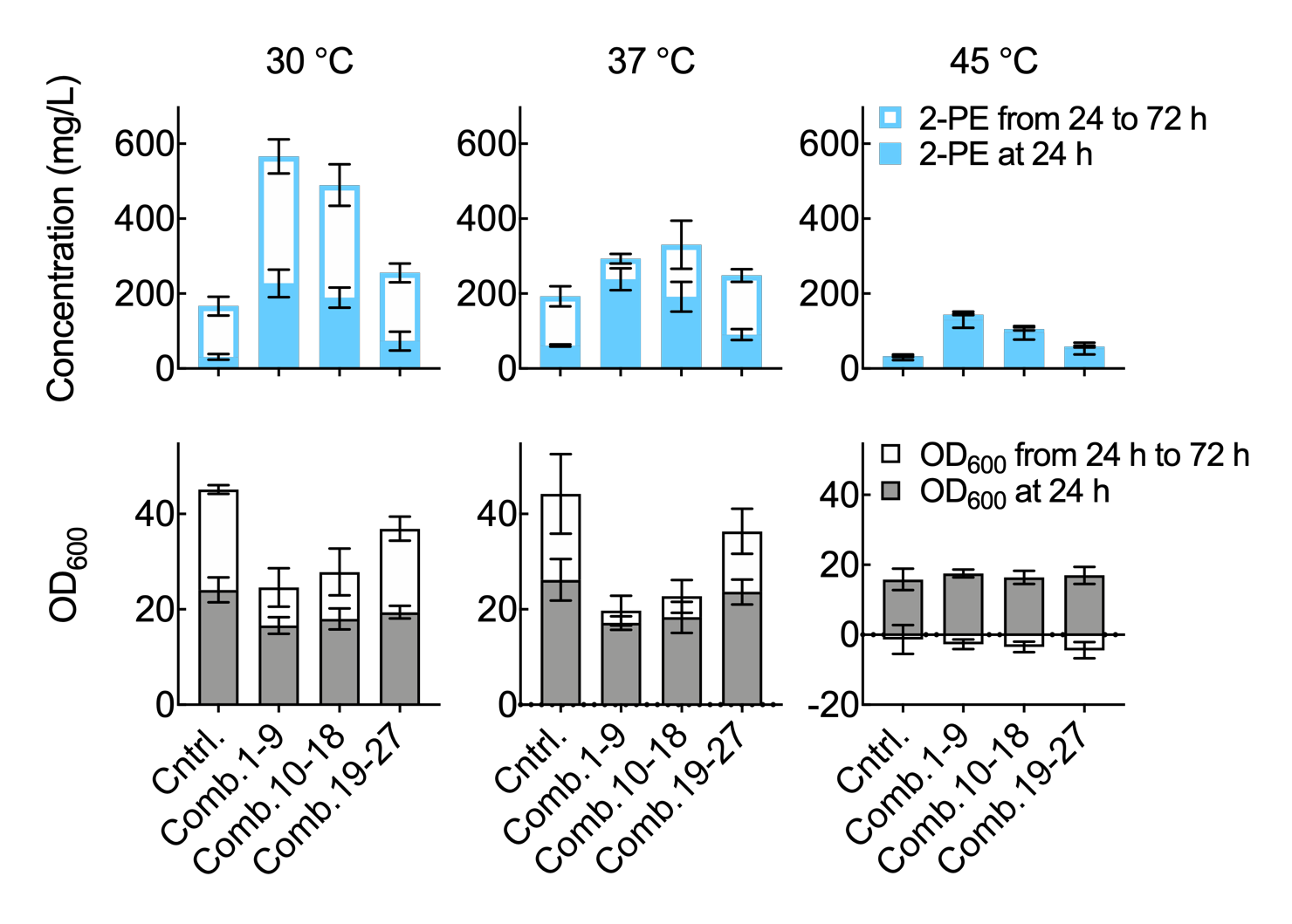


Figure S7. Temperature effects on 2-PE formation and biomass accumulation. The 2-PE concentration and biomass denoted by OD_600_ after 24 h cultivation, and their increase from 24 to 72 h were depicted at 30, 37, and 45 °C. Strains were cultivated in 25 mL YPD medium with 20 g/L D-glucose from an initial OD_600_ of 0.05. The 27 engineered strains were grouped into three subsets based on the overexpression level of *KmARO4^K221L^*. The strain Cntrl. was *K. marxianus* CBS 6556 *ura3Δ his3Δ* as the negative control. Bars are arithmetic means of at least triplicate biological samples, and error bars are standard deviations.


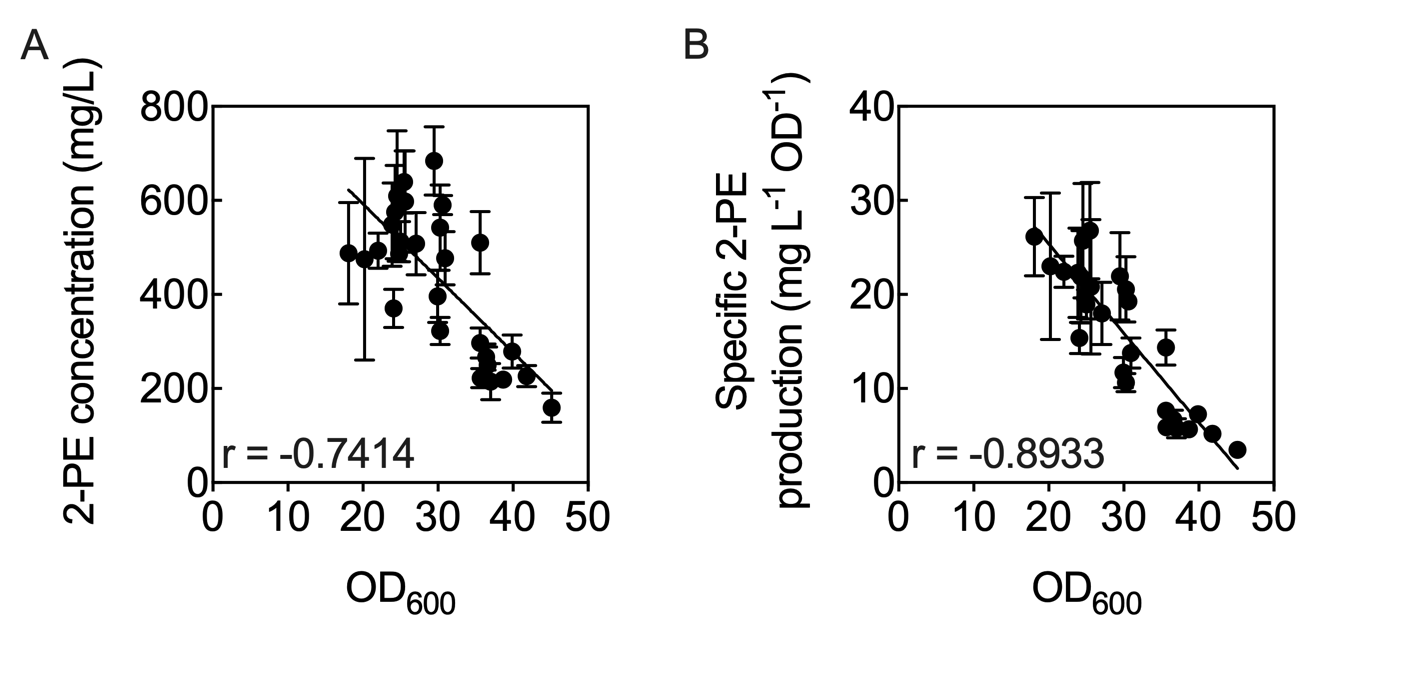


Figure S8. Correlation of the (A) 2-PE concentration and (B) specific 2-PE production with biomass accumulation (shown by OD_600_) after 72 h cultivation at 30 °C. Cell cultures were initiated from an OD_600_ of 0.05 in 25 mL YPD medium with 20 g/L D-glucose. The linear correlation of the two variables in both (A) and (B) are measured by the Pearson correlation coefficient, r.


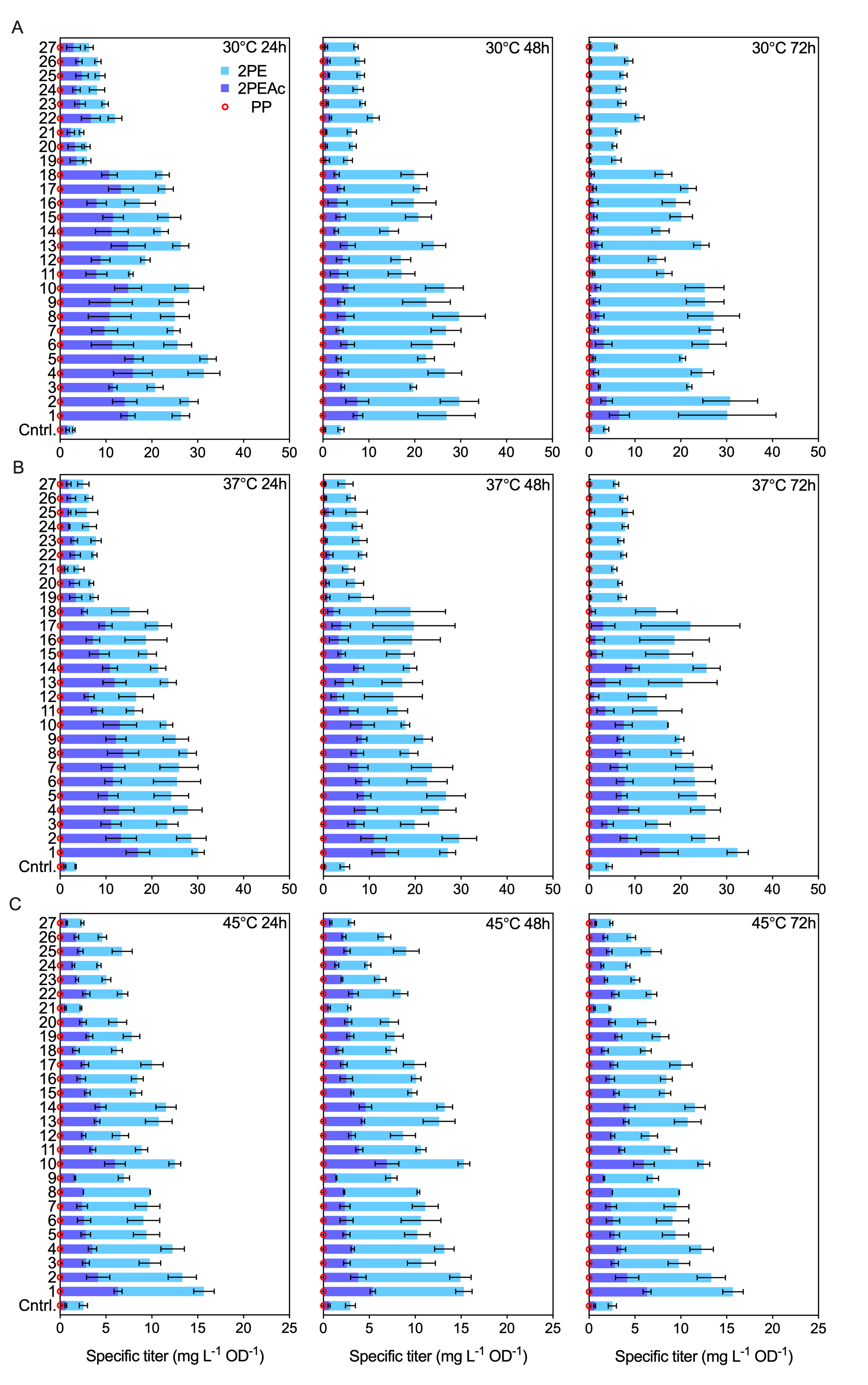


Figure S9. Specific production (mg L^-1^ OD^-1^) of 2-PE, 2-PEAc, and PP after 24, 48 and 72 h cultivation at (A) 30, (B) 37, and (C) 45 °C. Cell cultures were initiated from an OD_600_ of 0.05 in 25 mL YPD medium with 20 g/L glucose. The strain Cntrl. was *K. marxianus* CBS 6556 *ura3Δ his3Δ* as the negative control. Numbers 1 to 27 denoted corresponding gene overexpression combinations integrated into the genome of Cntrl. All experiments are performed at least in biological triplicates. Bars and dots represent arithmetic means, and error bars represent standard deviations.


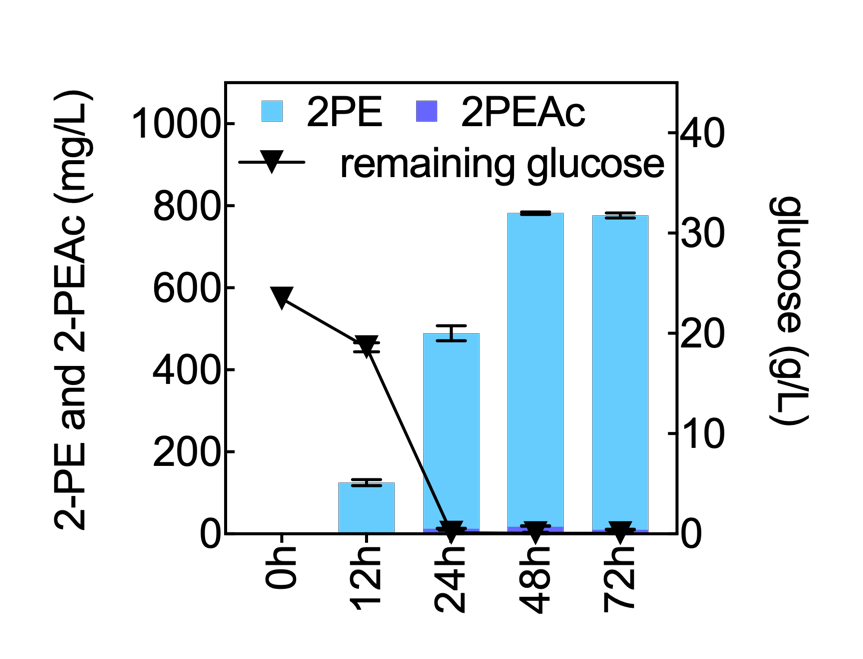


Figure S10. Extracellular 2-PE and 2-PEAc formation with glucose consumption in *K. marxianus* CBS 6556 *his3Δ* *eat1Δ* *ura3Δ*::(P*_KmTEF3_*)*KmARO10*, *abz1*::(P*_KmTEF3_*)*KmARO4^K221L^*-(P*_KmPGK_*)*KmARO7^G141S^*-(P*_KmTDH3_*)-*KmPHA2*. The strain was cultivated at 30 °C for 72 h in 25 mL YPD with 20 g/L D-glucose from an initial OD_600_ of 0.05. Bars and dots represent arithmetic means, and error bars represent standard deviations.


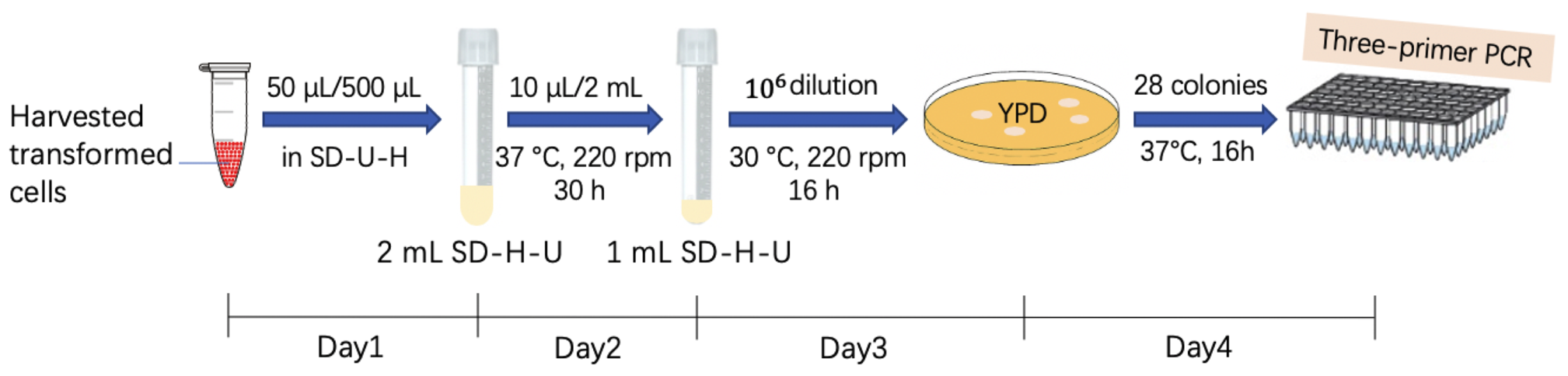


Figure S11. Schematic representation of the standard protocol for CRISPR-Cas9 mediated one-step integration up to three genes through yeast transformation to integration confirmation.


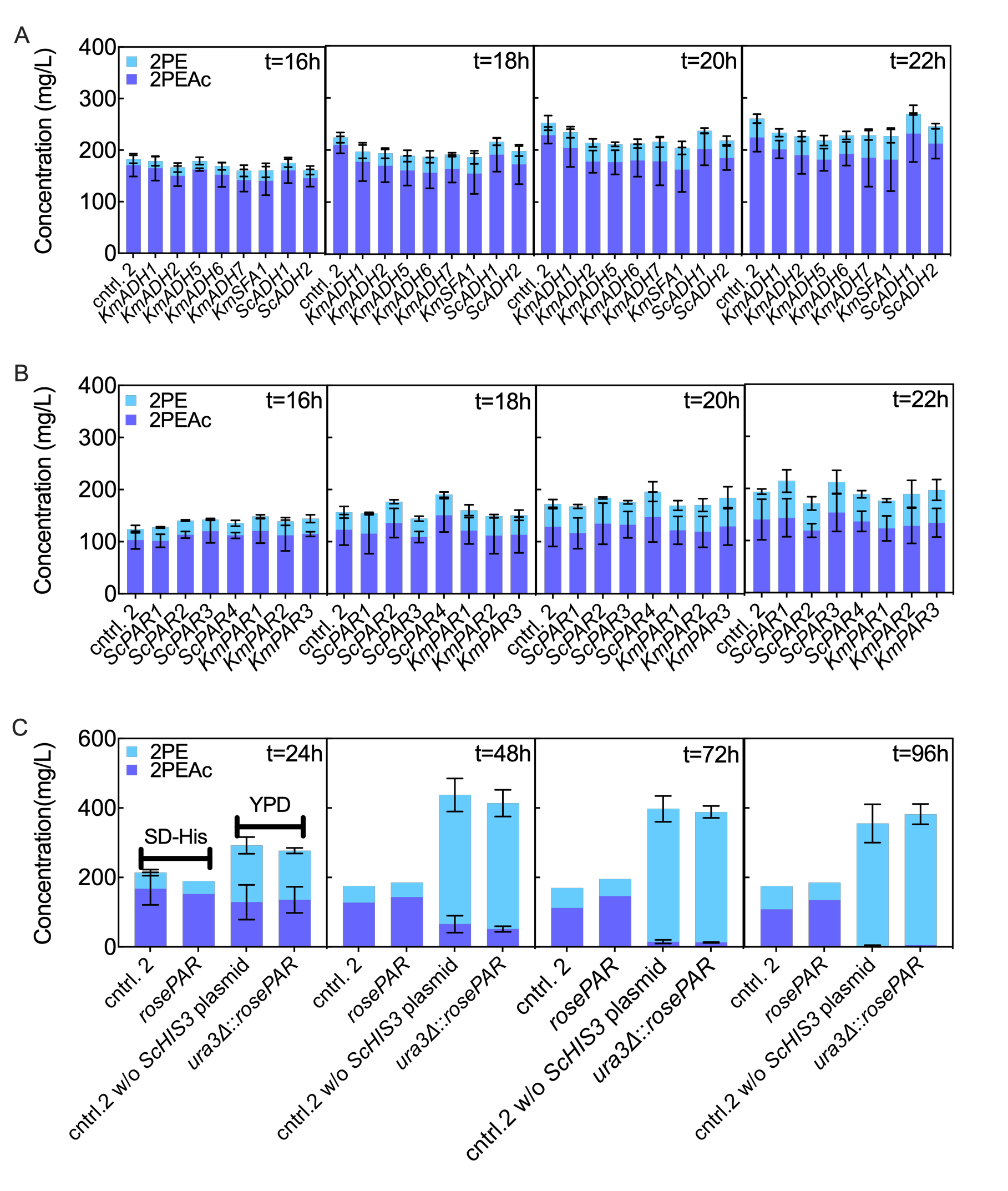


Figure S12. Effects of plasmid-based or chromosomal overexpression of homologous and heterologous genes encoding alcohol dehydrogenases (Adh) or phenylacetaldehyde reductases (Par) on 2-PE and 2-PEAc biosynthesis. The strain cntrl. 2 was *K. marxianus* CBS 6556 *ura3Δ his3Δ* *abz1*::(P*_ScTDH3_*)*KmARO4^K221L^*-(P*_ScTDH3_*)*KmARO10* harboring a blank vector expressing *ScHIS3*. Both selective medium SD-H and rich medium YPD contained 20 g/L D-glucose in the beginning of yeast cultivation with an OD_600_ of 0.05. Bars represent arithmetic means, and error bars represent standard deviation.


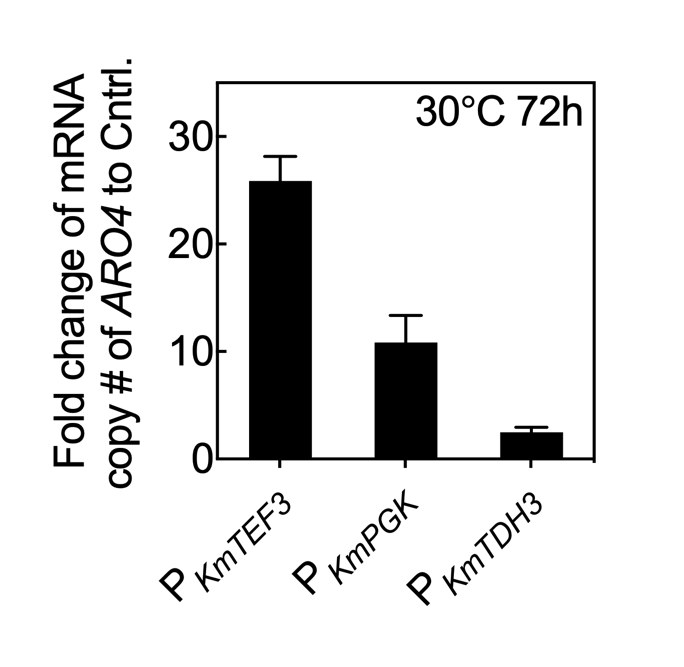


Figure S13. Relative transcriptional activity of the 700-bp natively derived *KmTEF3*, *KmPGK*, and *KmTDH3* promoter for *KmARO4* (including wild-type *KmARO4* and *KmARO4^K221L^*) expression. The strain Cntrl. was *K. marxianus* CBS 6556 *ura3Δ his3Δ*. mRNA was isolated after 72 h cultivation in 25 mL YPD medium with 20 g/L D-glucose at 30 °C.


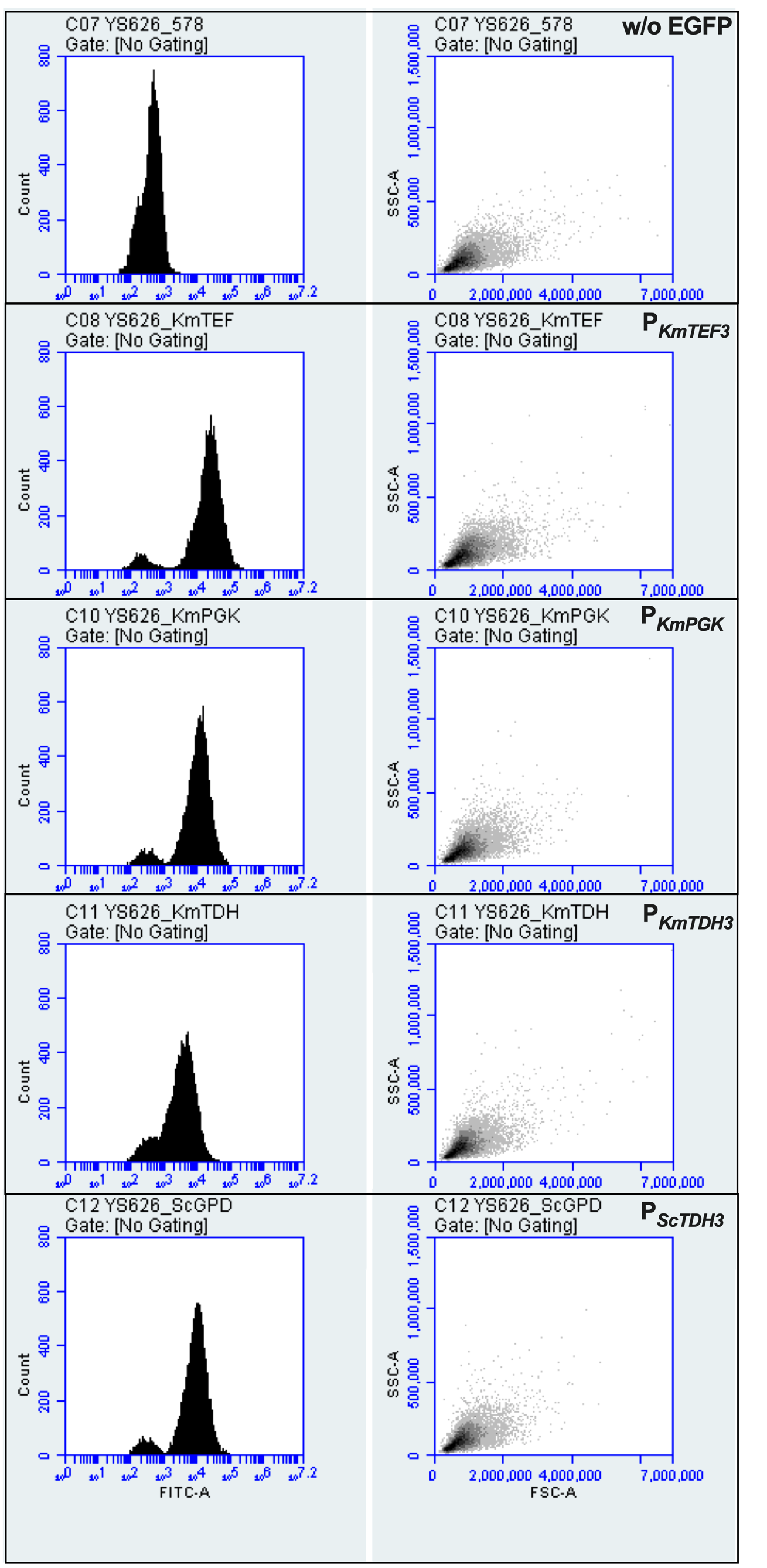


Figure S14. Histogram events of EGFP expression driven by P*_KmTEF3_*, P*_KmPGK_*, P*_KmTDH3_*, and P*_ScTDH3_* on plasmid at 30 °C, and corresponding cell size distribution.


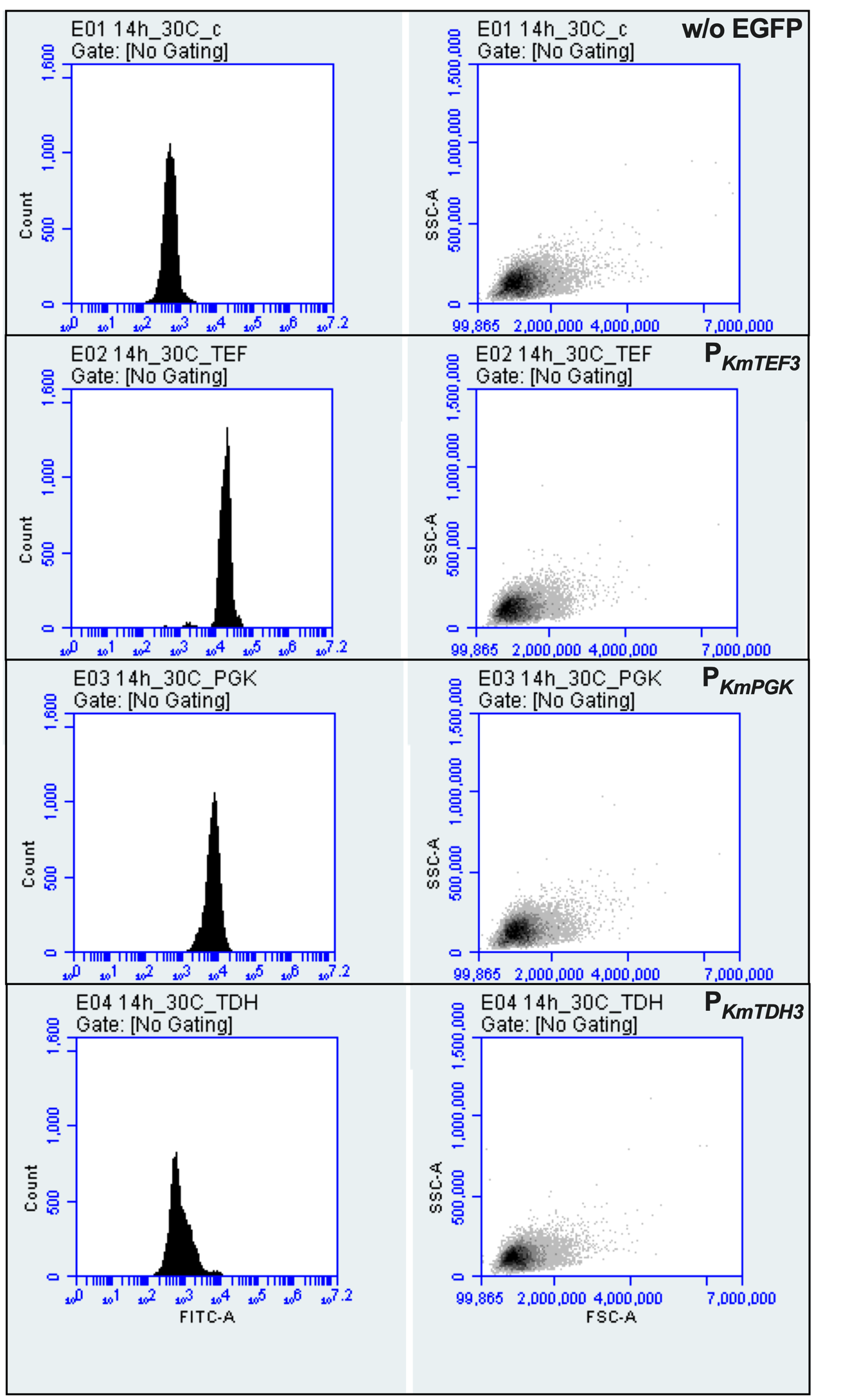


Figure S15. Histogram events of integrated EGFP expression driven by P*_KmTEF3_*, P*_KmPGK_*, and P*_KmTDH3_* at 30 °C, and corresponding cell size distribution.


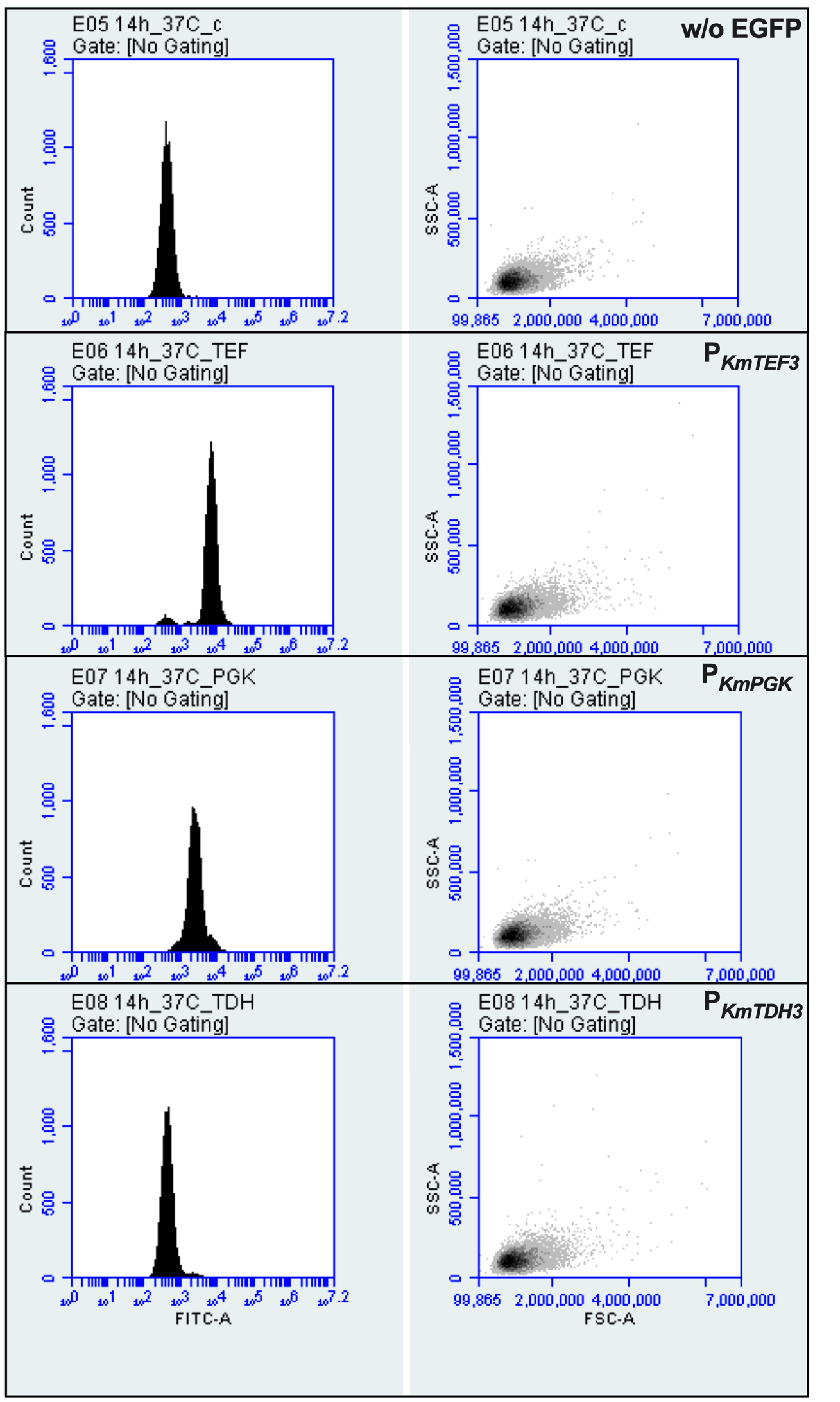


Figure S16. Histogram events of integrated EGFP expression driven by P*_KmTEF3_*, P*_KmPGK_*, and P*_KmTDH3_* at 37 °C, and corresponding cell size distribution.


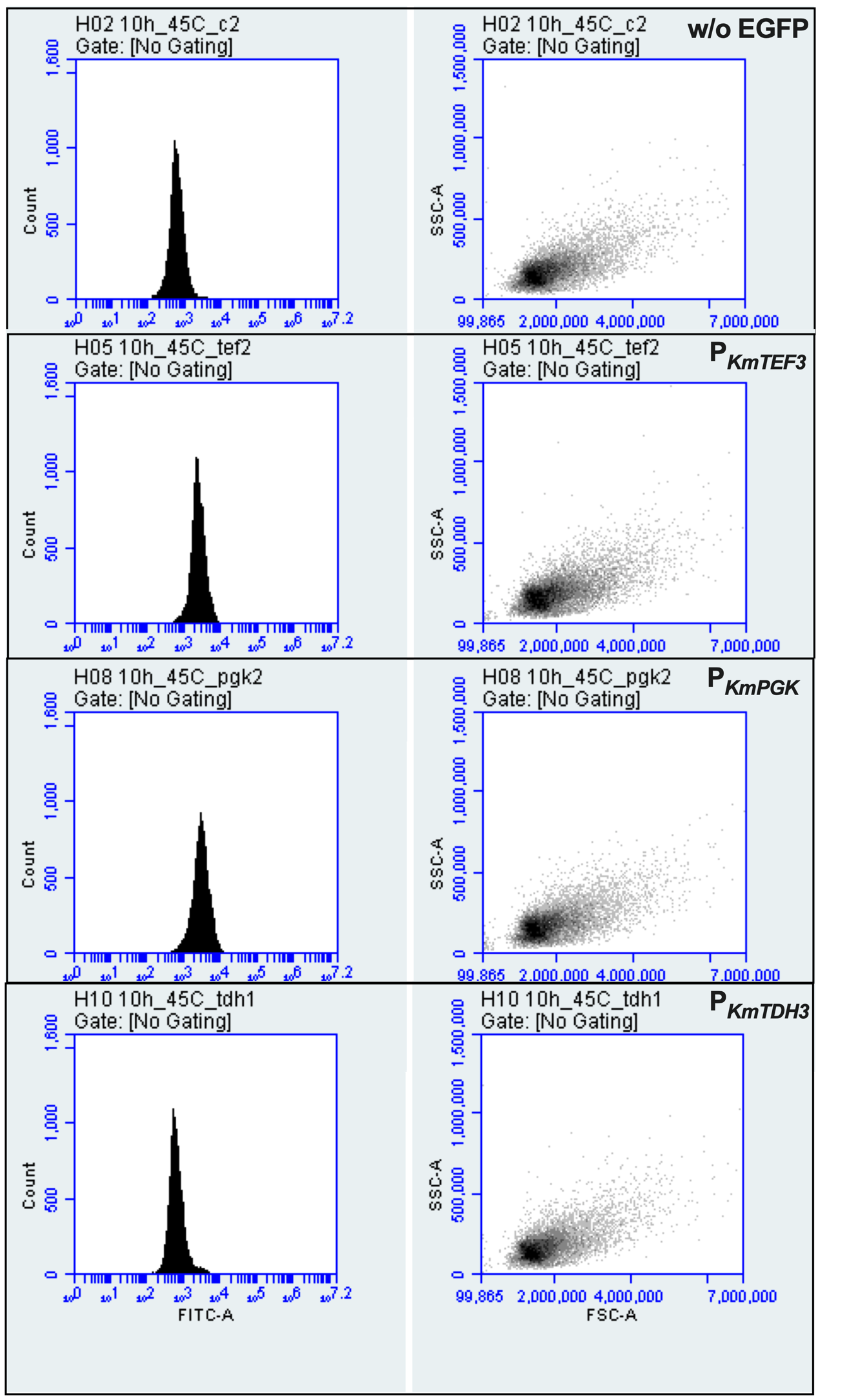


Figure S17. Histogram events of integrated EGFP expression driven by P*_KmTEF3_*, P*_KmPGK_*, and P*_KmTDH3_* at 45 °C, and corresponding cell size distribution.


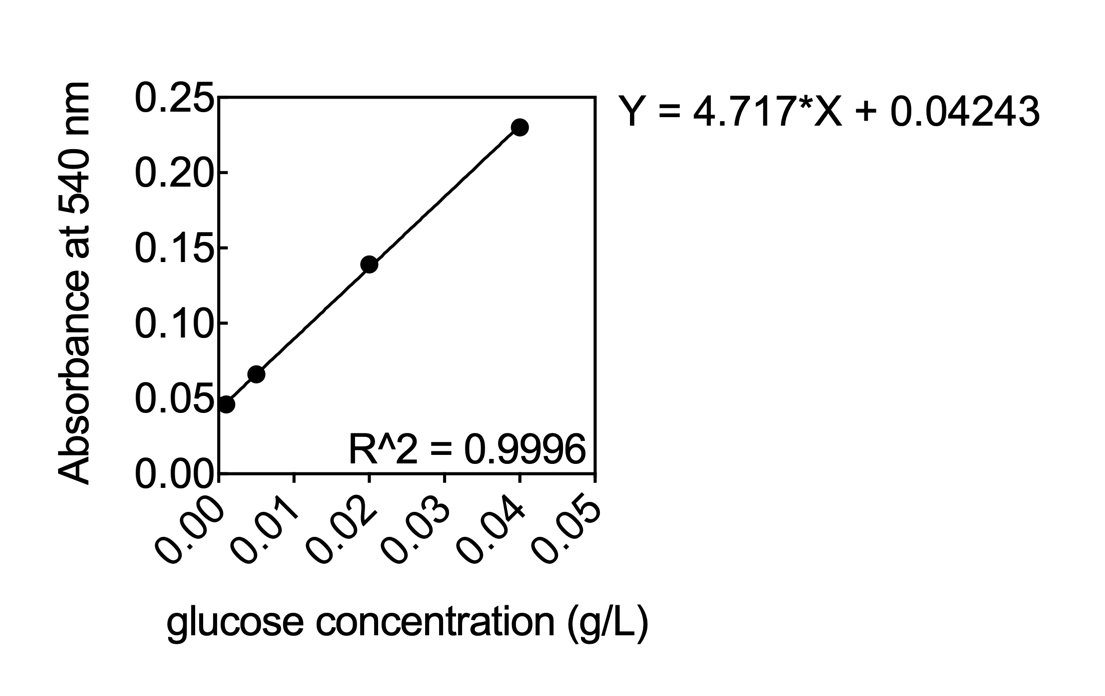


Figure S18. Standard curve of glucose concentration (g/L) to absorbance at 540 nm. 0.005 to 0.04 g/L of glucose solution is in the linear correlation region with the absorbance.

**Reference**

[1] Löbs, A.K., Engel, R., Schwartz, C. Flores, A., & Wheeldon, I. (2017). CRISPR–Cas9-enabled genetic disruptions for understanding ethanol and ethyl acetate biosynthesis in Kluyveromyces marxianus. *Biotechnology for biofuels*,*10*(1), 164. https://doi.org/10.1186/s13068-017-0854-5

[2] Lee, K-S., Kim, J-S., Heo, P., Yang, T-J., Sung, Y-J., Cheon, Y., Koo, H. M., Yu, B. J., Seo, J-H., Jin, Y-S., Park, J. C., & Kweon, D-H. (2013). Characterization of Saccharomyces cerevisiae promoters for heterologous gene expression in Kluyveromyces marxianus. *Applied microbiology and Biotechnology, 97*(5), 2029–2041. https://doi.org/10.1007/s00253-012-4306-7

[3] Zhu, J., Lin, J-L., Palomec, L., & Wheeldon, I*.* (2015). Microbial host selection affects intracellular localization and activity of alcohol-O-acetyltransferase. *Microbial cell factories,* *14*(1), 35. https://doi.org/10.1186/s12934-015-0221-9

[4] Lin, J-L., Zhu, J. & Wheeldon, I. (2017). Synthetic Protein Scaffolds for Biosynthetic Pathway Colocalization on Lipid Droplet Membranes. *ACS Synthetic Biology, 6*(8), 1534-1544. https://doi.org/10.1021/acssynbio.7b00041

[5] Löbs, A.K., Schwartz, C., Thorwall, S., & Wheeldon, I. (2018). Highly Multiplexed CRISPRi Repression of Respiratory Functions Enhances Mitochondrial Localized Ethyl Acetate Biosynthesis in Kluyveromyces marxianus. *ACS Synthetic Biology, 7*(11), 2647‐2655. https://doi.org/10.1021/acssynbio.8b00331

[6] Shao, Y., Lu, N., Wu, Z., Cai, C., Wang, S., Zhang, L-L., Zhou, F., Xiao, S., Liu, L., Zeng, X., Zheng, H., Yang, C., Zhao, Z., Zhao, G., Zhou, J-Q., Xue, X., Qin, Z. (2018). Creating a functional single-chromosome yeast. *Nature 560*(7718), 331–335. https://doi.org/10.1038/s41586-018-0382-x

[7] Lang, X., Besada-Lombana, P. B., Li, M., Da Silva, N. A., & Wheeldon, I. (2020). Developing a broad-range promoter set for metabolic engineering in the thermotolerant yeast Kluyveromyces marxianus. *Metabolic Engineering Communications*. Accepted.
